# Mediterranean diet and risk of prostate cancer: a systematic review and meta-analysis of observational studies

**DOI:** 10.1186/s40795-026-01544-5

**Published:** 2026-11-04

**Authors:** Tom Thabo Jahnke, Christian Holschuh, Peter Schlattmann

**Affiliations:** Institute of Medical Statistics, Computer Sciences and Documentation, Jena University Hospital, Friedrich Schiller University Jena, Jena, Germany

**Keywords:** Prostate cancer, Mediterranean diet, Nutrition, Primary prevention, Meta-analysis, Risk reduction, Public health

## Abstract

**Background:**

Prostate cancer is the most common malignancy among men worldwide. In addition to non-modifiable risk factors, modifiable lifestyle factors are increasingly recognized in prevention. The Mediterranean diet (Med Diet), characterized by high intake of plant-based foods, unsaturated fatty acids, and antioxidant micronutrients, has been proposed as a protective dietary pattern.

**Methods:**

A systematic search was conducted in PubMed, Web of Science, and Scopus examining associations between Mediterranean diet adherence and prostate cancer risk. Fourteen studies met eligibility criteria. Meta-analysis was performed in R applying a random-effects model (REML estimation; Hartung–Knapp adjustment). Heterogeneity was assessed with I² and τ²; publication bias was evaluated using a funnel plot, contour-enhanced funnel plot, and Egger’s test.

**Results:**

Based on a random effects model, we did not find a statistically significant difference in risk of prostate cancer in men with high versus low Mediterranean diet adherence (RR = 0.98; 95% CI: 0.93–1.04). Heterogeneity was moderate (Q = 19.95, I² = 34.8%). Although Egger’s regression test did not indicate statistically significant publication bias (*p* = 0.115), complementary analyses (Q-Q plot and limit meta-analysis) suggested a possible small-study effect, warranting cautious interpretation.

**Conclusion:**

In this meta-analysis, no protective effect of the Mediterranean diet against prostate cancer was found. Nevertheless, biological plausibility and inconsistent epidemiological trends suggest that further research using standardized dietary assessments and incorporating genetic and microbiome-related factors is needed.

**Trial registration:**

The review protocol was not prospectively registered.

**Supplementary Information:**

The online version contains supplementary material available at https://doi.org/10.1186/s40795-026-01544-5.

## Introduction

### Background

Prostate cancer is the most common malignant tumor among men worldwide and a leading cause of cancer-related mortality. According to the GLOBOCAN 2024 (Global Cancer Observatory 2024) estimates, approximately 1.55 million new prostate cancer cases and almost 420,000 deaths occurred worldwide in 2024, making prostate cancer the second most commonly diagnosed cancer among men globally [1].

While non-modifiable risk factors, such as age, genetic predisposition, and ethnicity, play a substantial role in disease incidence [2, 3], modifiable determinants are increasingly relevant in both prevention and research. These include primarily dietary behavior, body weight, and physical activity [4, 5]. Dietary factors have long been considered potential modifiable determinants of prostate cancer risk. Numerous epidemiological studies have investigated associations between dietary patterns, food groups, and individual nutrients with prostate cancer incidence, although findings have often been inconsistent. Diets characterized by high intakes of processed meat, saturated fats, and ultra-processed foods have been associated with increased inflammatory and metabolic disturbances, whereas plant-rich dietary patterns have been hypothesized to exert protective effects through anti-inflammatory and antioxidant mechanisms. The Mediterranean diet is characterized by high consumption of vegetables, fruits, legumes, whole grains, fish, nuts, and olive oil as the primary source of vegetable fat. Furthermore, it involves low intakes of red meat, sugar, ultra-processed foods, and moderate amounts of dairy and alcohol. Numerous studies indicate that greater adherence to this dietary pattern is associated with a reduced risk of multiple cancer types [6]. Several biological mechanisms have been proposed to explain the potential protective effects of the Mediterranean diet. These nutrient-dense components provide a range of antioxidants and other bioactive compounds that may contribute to the potential health effects of the Mediterranean diet. Furthermore, the diet may improve insulin sensitivity, influence insulin-like growth factor signaling, and promote a more diverse gut microbiome, all of which have been implicated in carcinogenesis [7–10]. Given these biological and epidemiological considerations, synthesizing the currently available evidence is essential to better understand whether adherence to the Mediterranean diet is associated with prostate cancer risk.

### Scientific context

Although diet and lifestyle have been extensively studied in the context of prostate cancer etiology [11], findings remain inconsistent. According to the 2018 WCRF/AICR Continuous Update Project Report, the evidence linking dietary patterns, including adherence to the Mediterranean diet, to prostate cancer risk was considered insufficient to support definitive conclusions [5].

While some previously published large cohort studies, such as the EPIC analysis by Couto et al., suggest a potential protective association between Mediterranean dietary patterns and overall cancer risk [12], other large studies found no significant evidence of a protective effect [13, 14], thereby providing a rationale for updated meta-analyses.

Additionally, the Mediterranean diet has been associated with reduced risk of cardiovascular disease, metabolic syndrome, as well as several other types of cancer, including colorectal and breast cancer [15–20]. Nevertheless, evidence regarding prostate cancer remains unclear, partly due to variation in dietary assessment tools, study populations, and statistical models, contributing to heterogeneity and inconsistent findings.

### Research gap and relevance

The most comprehensive analysis to date, published by Cheng et al. [21], found no risk reduction, but was limited by a small number of studies and methodological constraints. Therefore, it is advisable to further evaluate this topic to draw definite conclusions.

Given the high global prevalence of prostate cancer, dietary interventions could provide a low-threshold intervention and may be relevant for cancer prevention, including overdiagnosis in prostate cancer screening.

### Study objective

Therefore, this systematic review and meta-analysis aimed to comprehensively evaluate the association between adherence to the Mediterranean diet and prostate cancer risk by synthesizing the currently available observational evidence. Furthermore, we sought to explore potential sources of heterogeneity and assess the robustness and quality of the available evidence.

## Methods

### Search strategy

**Fig. 1:**
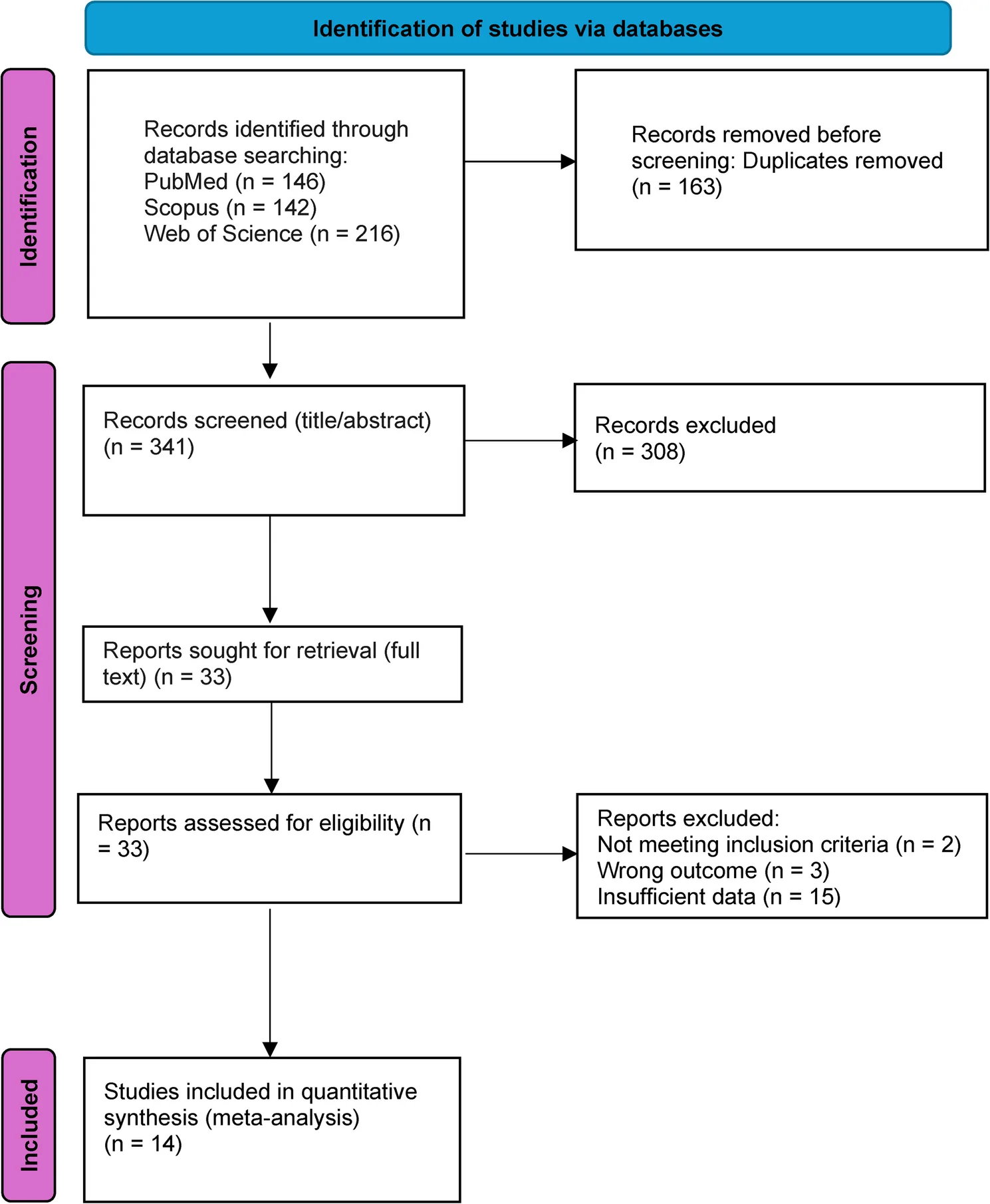
PRISMA 2020 flow diagram illustrating the study selection process. The figure depicts the number of records identified, screened, excluded, and included in the meta-analysis according to PRISMA guidelines. Adapted from Page et al. [22]

This systematic review and meta-analysis was conducted in accordance with the PRISMA guidelines [22]. A systematic literature search was performed in PubMed, Web of Science, and Scopus using a predefined English-language search string to combine terms related to the Mediterranean diet and prostate cancer (see supplements), restricted to human studies only. The search covered studies published between January 1, 1995, and February 2026, to ensure a contemporary evidence base. No restrictions were imposed on study design at this stage. The search was last updated on February 12, 2026 (Fig. 1).

### Study selection

The initial literature search identified 504 records, comprising 146 from PubMed, 216 from Web of Science, and 142 from Scopus. After removing duplicates (*n* = 163) and conducting title and abstract screening (exclusion of Meta-analyses, Reviews or Guidelines, *n* = 80, studies that did not address prostate cancer, *n* = 16; studies that did not address Mediterranean diet or only partial aspects of it, *n* = 76; or did not meet either inclusion criterion, *n* = 130), 33 full-text articles were assessed for eligibility. Of these, 14 studies met all predefined inclusion criteria and were subsequently included in the meta-analysis. Multiple reasons for exclusion could apply to a single study. Eligible studies were required to be primary investigations with a prospective or retrospective design that examined the association between adherence to a Mediterranean dietary pattern and prostate cancer incidence. Additionally, studies had to include a clear, predefined assessment of Mediterranean diet exposure. Studies were also required to report effect estimates, including relative risks (RRs), odds ratios (ORs), or hazard ratios (HRs), along with corresponding 95% confidence intervals. Full-text availability in English was mandatory. Studies were excluded if they were reviews, meta-analyses, guidelines, editorials, or opinion papers. Studies focusing solely on isolated dietary components, such as lycopene or olive oil, were also not eligible. Further exclusion criteria included the absence of risk estimates, a sole focus on grade group or disease progression without addressing incidence, inadequate or missing control groups, and preclinical or animal-based study designs. Concerning the research question under consideration, no randomized controlled trials were available; therefore, this meta-analysis was limited exclusively to prospective cohort studies and case-control studies.

### Data extraction

Data from all included studies were extracted independently and then compiled into a standardized extraction table (Table 1). Study selection and data extraction were initially performed by the first author (TTJ) and independently verified by a second reviewer (CH). Any discrepancies were resolved through discussion until consensus was reached.

**Table 1:** Overview of included studies

Author (Year)	Country	Study design/ Population	Exposure (Score; comparison categories)	Outcome/ Follow-up	Effect estimate (95% CI)	Main adjustments
Schulpen et al. [52]	Netherlands	Cohort; *n* = 58,279; 3,868 cases; mean age 62	Alternate Mediterranean diet score without alcohol (aMEDr, ) and modified Mediterannean diet score (mMED) (questionnaire; tertiles of adherence; (0–3), (4–5), (6–8) and continuous per 2 pt increase )	PCa incidence; 20.3 years	1.09 (1.01–1.17)	age, BMI, energy intake, alcohol, family history, education
Ax et al. [45]	Sweden	Cohort; *n* = 566; 72 cases; mean age 71.0	mMDS (questionnaire; tertiles based on a score from 0–8; low (≤ 2 points), medium (3–5 points), or high (≥ 6 points))	PCa incidence; 13.3 years	1.04 (0.43–2.49)	age, education, physical activity, smoking, energy intake
Bosire et al. [13]	USA	Cohort; *n* = 293,464; 23,453 cases; age 50–71	aMED (questionnaire; ≤2, 3, 4, 5, and ≥ 6; quintiles of Diet Quality Scores) )	PCa incidence; 8.9 years	0.97 (0.91–1.03)	age, BMI, education, physical activity, smoking, ethnicity
Jalilpiran et al. [49]	Iran	Case–control; *n* = 60 cases, 60 controls; mean age 66	MD pattern (interview; score and cutoffs not available)	PCa incidence; not available	0.62 (0.22–1.77)	age, BMI, energy intake, physical activity, smoking, education, height, other drugs
Lavalette et al. [50]	France	Cohort; *n* = 41,543; 222 cases; mean age 61.6	MEDI-LITE (questionnaire; quintiles of adherence based on a score from 0–18; 1st quintile: 0–6; 2nd Quintile: 7–7; 3rd quintile: 8–9; 4th quintile: 10–10; 5th quintile: 11–16)	PCa incidence; 8 years	0.95 (0.61–1.50)	age, BMI, physical activity, energy intake, family history
Muller et al. [43]	Australia	Cohort; *n* = 14,627; 1,018 cases; age 34–75	Mediterranean diet score (MDS) (questionnaire; quartiles of adherence; cutoffs not available)	PCa incidence; 13.6 years	0.93 (0.74–1.18)	age, ethnicity, energy intake
Russo et al. [51]	Italy	Case–control; *n* = 118 cases; 238 controls; mean age 69.1	MEDI-LITE (interview; tertiles of adherence; low (1–3), medium (4–7), high (> 7) and continuous (one point increase) )	PCa incidence; not available	0.16 (0.03–0.72)	age, BMI, smoking
Kenfield et al. [14]	USA	Cohort; *n* = 47,867; 6,220 cases; age 40–75	aMED, MDS (questionnaire; tertiles of adherence (score 0–3), moderate adherence (score 4–5), high adherence (score 6–9)	PCa incidence & mortality; 23.3 years	0.95 (0.90–1.02)	age, BMI, energy intake, physical activity, smoking, diabetes, height, family history, PSA screening
Castello et al. [47]	Spain	Cohort; *N* = 1,277 cases; 754 controls; age 38–85	Principal component analysis-derived Mediterranean dietary pattern (interview; quartiles of diet quality scores; cutoffs not available)	PCa incidence; not available	0.90 (0.66–1.23)	age, BMI, family history, education, energy intake, residence
Möller et al. [44]	Sweden	Case-control; *n* = 1,482 cases; 1,108 controls; mean age 66.8/67.7	Mediterannean Diet Score (MDS) (questionnaire; tertiles of adherence; low (0–3), medium (4–5), high (6–9) )	PCa incidence; not available	1.03 (0.81–1.30)	age, BMI, family history, education, energy intake, residence, diabetes, smoking, physical activity
Askari et al. [55]	Iran	Case–control; *n* = 52 cases; 104 controls; age 40–78	Mediterannean style dietary pattern score (MSDPS) (interview; tertiles of adherence; 1st tertile 2.07–8.01; 2nd tertile 8.11–12.20; 3rd tertile 12.21–28.06 ; MSDPS Range Controls: 1st tertile 2.50-11.54; 2nd tertile 11.55–18.44; 3rd tertile 18.45–54.64)	PCa incidence; not available	OR 0.28 (0.08–0.91)	age, BMI, smoking, energy intake, education
Urquiza-Salvat et al. [53]	Spain	Case–control; *n* = 254 cases; *n* = 302 controls; age not available	Mediterranean diet score (MDS) (questionnaire; quartiles of adherence; cutoffs not available)	PCa incidence; not available	HR 0.98 (0.88–1.00)	age, total energy intake, BMI, smoking status, physical activity, education
Castello et al. [48]	Spain	Cohort; full sample *n* = 15,296; *n* = 609 cases; *n* = 14,687 controls; mean age 49.92 years	Principal component analysis-derived Mediterranean dietary pattern (interview; quartiles of Diet quality scores; cutoffs not available)	PCa incidence; 16.00 years	HR 1.05 (0.79–1.38)	age, center, energy intake, BMI, height, education
Lozano-Lorca et al. [54]	Spain	Case–control; full sample *n* = 827; *n* = 428 cases; *n* = 399 controls; mean age 68.5/ 66.5	Mediterranean pattern (questionnaire; tertiles of adherence; cutoffs not available)	PCa incidence; not available	OR 0.90 (0.63–1.29)	age, energy intake, BMI, smoking, education, family history

Characteristics of studies included in the meta-analysis Summary of study design, population characteristics, Mediterranean diet assessment methods, outcomes, and multivariable-adjusted effect estimates for the association between adherence to a Mediterranean dietary pattern and prostate cancer risk. Effect estimates refer to the comparison of the highest versus lowest category of adherence unless otherwise specified. Adjustments reflect the fully adjusted model reported by each study

*Abbreviations*: *CI* confidence interval, *RR* relative risk, *OR* odds ratio, *HR* hazard ratio, *PCa* prostate cancer, *BMI* body mass index, *aMED* alternate Mediterranean diet score, *mMED* modified Mediterranean diet score, *MDS* Mediterranean diet score, *MSDPS* Mediterranean-Style Dietary Pattern Score, *PCA* Principal component analysis

Extracted information included study characteristics such as author(s), year of publication, country or region, and study design. Population-related variables included total sample size and either the mean age or age range of the study participants. For exposure assessment, information was collected on the definition and measurement of adherence to a Mediterranean dietary pattern, including the Mediterranean Diet score described above. In addition, the categorization of dietary adherence, typically contrasting the highest versus the lowest categories, and the dietary assessment instruments used were recorded. In most studies, dietary intake was assessed using validated food frequency questionnaires (FFQs) [23, 24] administered during face-to-face interviews, validated self-administered questionnaires, telephone interviews, or interviewer-assisted questionnaires, depending on the individual study.

Outcome data included the risk of prostate cancer as the primary endpoint, with further differentiation by disease subtypes where available. Prostate cancer–specific mortality was also extracted when reported, but was not included in the pooled effect size. Information on covariate adjustment was recorded in detail, including adjustment for age, body mass index (BMI), family history of cancer, prostate-specific antigen (PSA) screening, physical activity, smoking status, alcohol consumption, total energy intake, and other relevant lifestyle-related factors.

When multiple statistical models were reported within a study, the model with the most comprehensive multivariable adjustment was selected for the meta-analysis. Hazard ratios were treated as equivalent to relative risks, and odds ratios were approximated as relative risks under the rare disease assumption. This approach is considered appropriate given the low incidence of prostate cancer in the underlying populations and is consistent with established methodological guidance [25]. Several indices were used across studies to operationalize adherence to the Mediterranean diet. The modified Mediterranean Diet score (mMED), originally proposed by Trichopoulou et al. [26] is based on sex-specific medians of key food groups, including vegetables, fruits, legumes, cereals, and fish, and assigns inverse scores for meat and dairy consumption. The alternate Mediterranean Diet score (aMED), developed by Fung et al. [27], was adapted to U.S. dietary habits by excluding dairy products, distinguishing between whole and refined grains, and assigning separate scores for processed meat and alcohol intake. The relative Mediterranean Diet score (rMED), introduced by Buckland et al. [28] uses tertile-based scoring rather than median cut-offs and adjusts for total energy intake, thereby improving comparability across countries in multi-center cohort studies.

### Statistical analysis

Statistical analyses were conducted in RStudio version 4.4.2 (R Foundation for statistical computing, Vienna, Austria) utilizing the RStudio integrated development environment (Posit, PBC, Boston, MA, USA) [29]. Meta-analytic procedures were performed using the packages meta (version 8.2-0) [30], metasens (version 1.5-3) [31], metafor (version 4.8-0) [32], and robvis (version 0.3.0) [33].

For meta-analysis, a random-effects model was used, and between-study variance (τ²) was estimated with Restricted Maximum Likelihood (REML) [32, 34]. Given the moderate number of included studies, Hartung–Knapp adjustments [35] were applied to produce more conservative confidence intervals.

### Sensitivity analyses according to exposure operationalization and study design

To assess whether study design influenced the pooled association, sensitivity analyses were performed separately for cohort and case-control studies using the same random-effects model as the primary analysis, with restricted maximum likelihood estimation of between-study variance and Hartung-Knapp adjustment of the confidence intervals. Differences between study-design subgroups were assessed using a formal test for subgroup differences. Because the included studies used heterogeneous approaches to operationalize Mediterranean diet adherence, a further exploratory sensitivity analysis was performed according to the underlying method of exposure assessment. Studies were categorized as using either predefined/index-based Mediterranean diet measures (e.g., aMED, mMDS, MEDI-LITE, MDS, or MSDPS) or PCA/factor-derived Mediterranean dietary patterns. Separate random-effects meta-analyses were conducted for each category using restricted maximum-likelihood estimation of between-study variance and Hartung–Knapp adjustment. A formal test for subgroup differences was used to assess whether pooled effect estimates differed between exposure-operationalization categories. Because individual Mediterranean diet instruments were generally represented by only one or a small number of studies, instrument-specific meta-analyses were not considered sufficiently informative.

### Heterogeneity and publication bias

Cochran’s Q test was used to evaluate heterogeneity across studies [36]. Additionally, the I² statistic quantified the proportion of total variability attributable to between-study differences rather than chance [37, 38]. Moreover, the between-study variance (τ²) was estimated to provide an absolute measure of heterogeneity on the log-risk-ratio scale.

Given Cochran’s Q test’s limited power, especially with few studies, a p-value below 0.10 was considered significant.

Publication bias and small-study effects were analyzed using both funnel-plot and contour-enhanced funnel-plot visualizations, as well as formal tests. Egger’s regression test was applied to detect funnel plot asymmetry [39].To evaluate the robustness of pooled results and address small-study effects, a limit meta-analysis and Q-Q′ test were conducted [40, 41].

To further explore sources of heterogeneity, a meta-regression was conducted to examine whether study design or geographic region influenced the association between Mediterranean diet adherence and prostate cancer risk. Furthermore, a leave-one-out sensitivity analysis was performed, removing each study in turn to assess its impact on the overall effect estimate [32, 34], with results provided in the supplementary material (see Supplements 1).

### Risk of bias assessment

The ROBINS-E tool (Risk of Bias in Non-randomized Studies of Exposure tool) [42] was used to assess the risk of bias. A detailed evaluation of the included studies in all seven domains is provided in the supplements (see Supplements 1).

Overall, the methodological quality of the included studies was judged to fall victim to some concerns. Most studies were rated as having either a high risk of bias or some concerns, primarily due to limitations in dietary exposure assessment, which relied predominantly on food frequency questionnaires and self-reported dietary intake.

## Results

### Literature search and study characteristics

The 14 included studies [13, 14, 43–55] were published between 2009 and 2023 (Table 1). They include both cohort and case–control designs and originate from Europe, North America, and Asia. The size of the study populations varied in size from 120 to 293,464 participants, with ages spanning from 38 to 85 years. The follow-up period, where available, ranged from 8 to 23 years. In most cases, the key confounders adjusted for were age, BMI, level of education, PSA screening behavior, family history, and socioeconomic factors, although the adjustment methods varied considerably.

The included studies reported effect estimates, including relative risks (RRs), hazard ratios (HRs), and odds ratios (ORs). For quantitative synthesis, all effect measures were pooled on a logarithmic scale.

### Meta-analysis: Mediterranean diet and prostate cancer risk

**Fig. 2:**
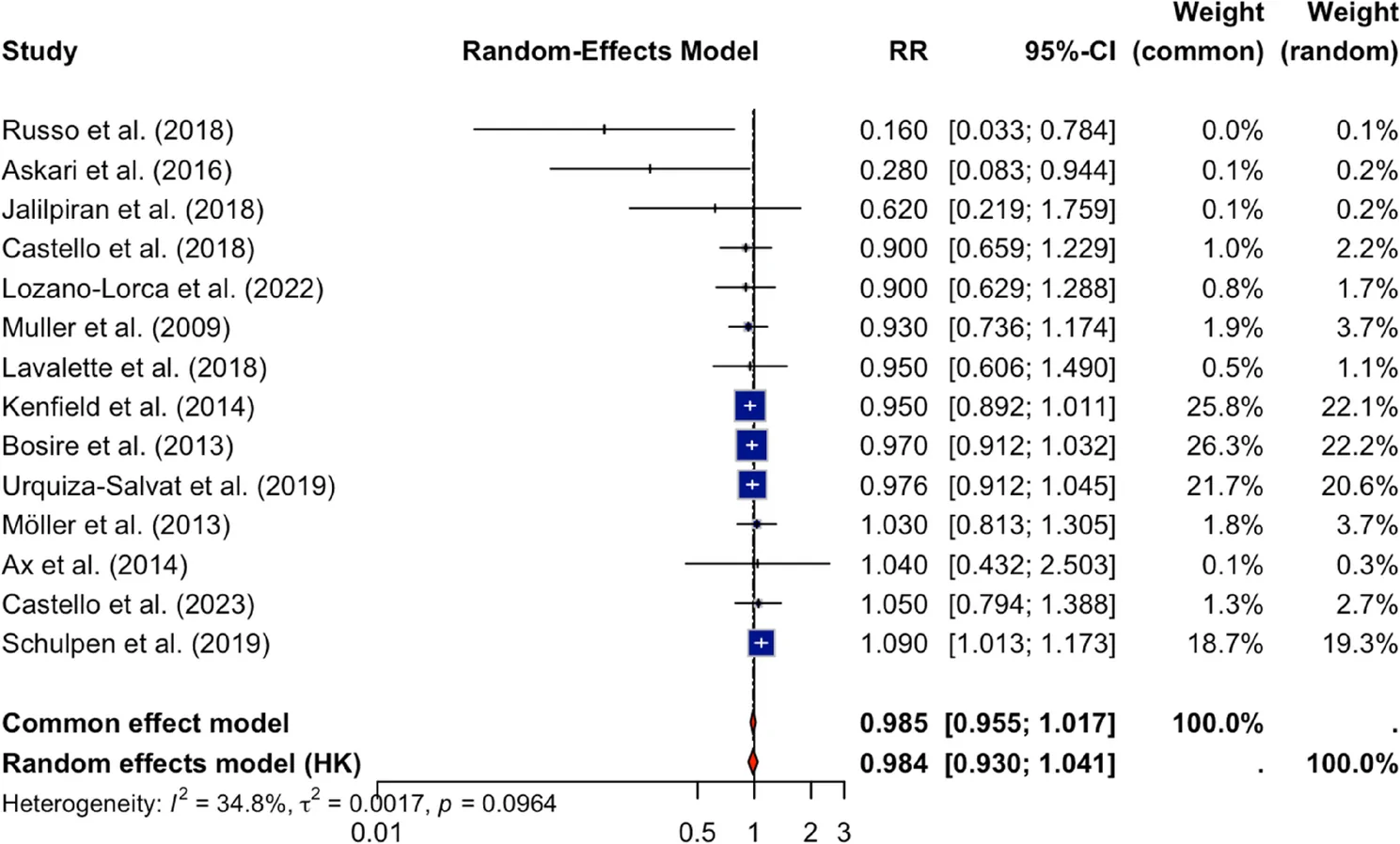
Forest plot of included studies assessing the association between the Mediterranean diet and prostate cancer risk. Forest plot showing the study-specific and pooled relative risks (RR) with 95% confidence intervals. Heterogeneity among studies is quantified by τ², τ, and I². The pooled estimate reflects the overall effect across all studies.

Under a random-effects model, the pooled effect of the 14 included studies yielded a relative risk (RR) of 0.984 (95% CI: 0.930–1.041; *p* = 0.55), indicating no statistically significant association between adherence to the Mediterranean diet and prostate cancer incidence. A corresponding forest plot is shown in Fig. 2. The common-effect model provided a similar estimate (RR = 0.985; 95% CI: 0.955–1.017; *p* = 0.36). Given heterogeneity across study designs and populations, the random-effects estimate is considered more appropriate for interpretation.

### Heterogeneity

Between-study heterogeneity was moderate, with I² = 34.8% and a between-study variance of τ² = 0.0017 (τ = 0.0409). Using α = 0.10 as a threshold for heterogeneity testing, the Q test [36] indicated borderline heterogeneity (Q = 19.95; df = 13; *p* = 0.0964). The H statistic was 1.24 (95% CI: 1.00-1.70), indicating that the observed variability exceeded sampling error by 24%.

### Meta-regression and sensitivity analyses

Since a certain degree of heterogeneity was observed, meta-regression was conducted to assess whether study design or geographical region influenced effect estimates. Study type was coded dichotomously for both parameters (case-control vs. cohort studies; Mediterranean vs. non-Mediterranean region). No statistically significant moderating effect in cohort studies compared to case-control studies was observed (β = 0.031; 95% CI 0.078 to -0.142; *p* = 0.58). Residual heterogeneity remained moderate (τ² = 0.0023; I² = 29.98%), and the covariate explained none of the between-study variability (R² = 0.00%). Given the limited number of studies, this finding should not be interpreted as evidence that study design is unimportant as a source of heterogeneity.

Similarly, geographical region did not significantly influence the pooled effect estimate (β = −0.027; 95% CI − 0.084 to 0.153; *p* = 0.573). Residual heterogeneity persisted (τ² = 0.0023; I² = 29.68%), and no heterogeneity was explained by the regional classification (R² = 0.00%). An overview of the meta-regression results is provided in the supplements (See Supplements 1 Table S3). In further sensitivity analyses stratified by study design, the pooled effect estimate was 0.992 (95% CI 0.939–1.048; *p* = 0.734) among the eight cohort studies and 0.969 (95% CI 0.860–1.093; *p* = 0.534) among the six case-control studies. Heterogeneity was 26.4% among cohort studies and 50.5% among case-control studies. There was no statistically significant difference between the pooled estimates according to study design (Q = 0.19, df = 1, *p* = 0.661).

### Sensitivity analysis according to exposure operationalization

To explore whether differences in the operationalization of Mediterranean diet adherence contributed to variability in the pooled association, we performed an exploratory subgroup analysis distinguishing predefined/index-based measures from PCA/factor-derived dietary patterns. Among the nine studies using predefined/index-based measures, the pooled effect estimate was 0.989 (95% CI 0.914–1.071; *p* = 0.765), with moderate heterogeneity (I² = 56.0%; τ² = 0.0021). Among the five studies using PCA/factor-derived patterns, the pooled estimate was 0.941 (95% CI 0.838–1.057; *p* = 0.221), with no statistical evidence of heterogeneity (I² = 0%; τ² = 0). Although the pooled estimate was numerically lower in the PCA/factor-derived subgroup, the formal test for subgroup differences was not statistically significant (Q = 0.85, df = 1; *p* = 0.356).

### Publication bias and limit meta-Analysis

**Fig. 3:**
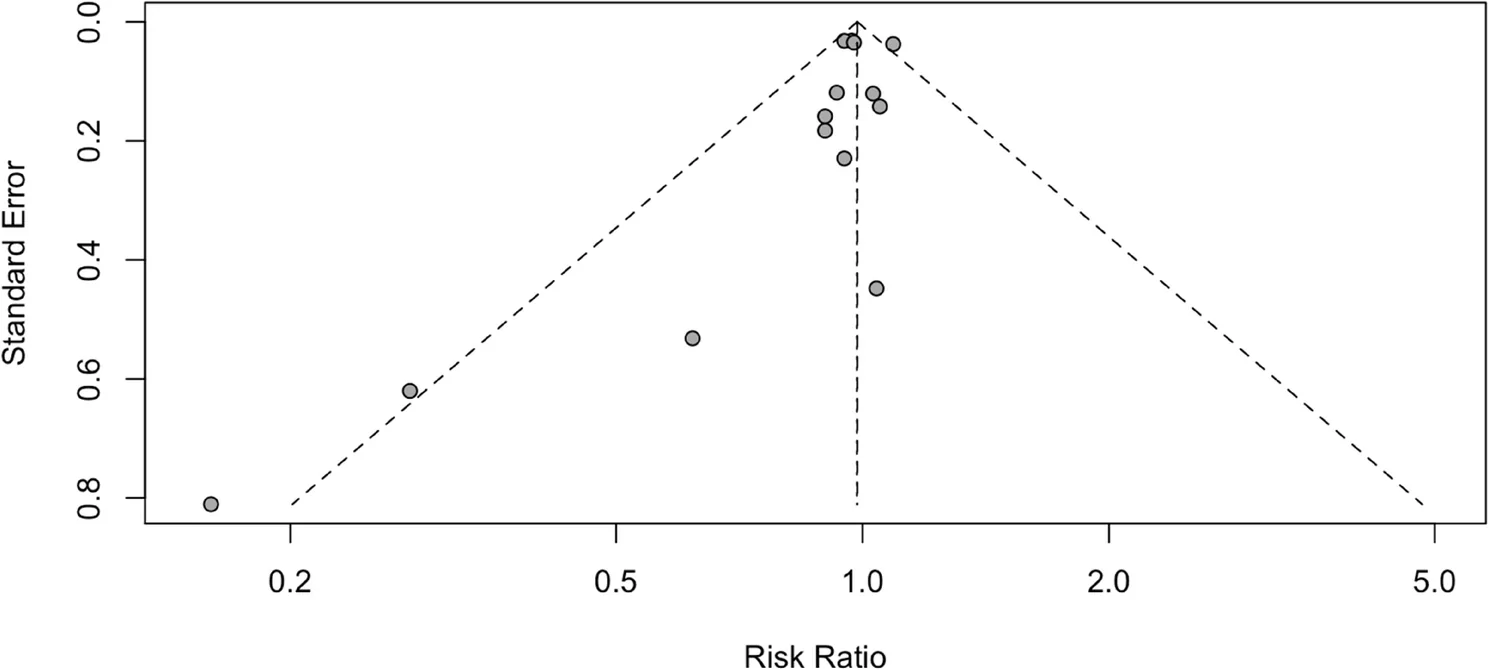
Funnel plot for the assessment of publication bias. Standard errors of log-transformed risk estimates plotted against effect sizes

**Fig. 4:**
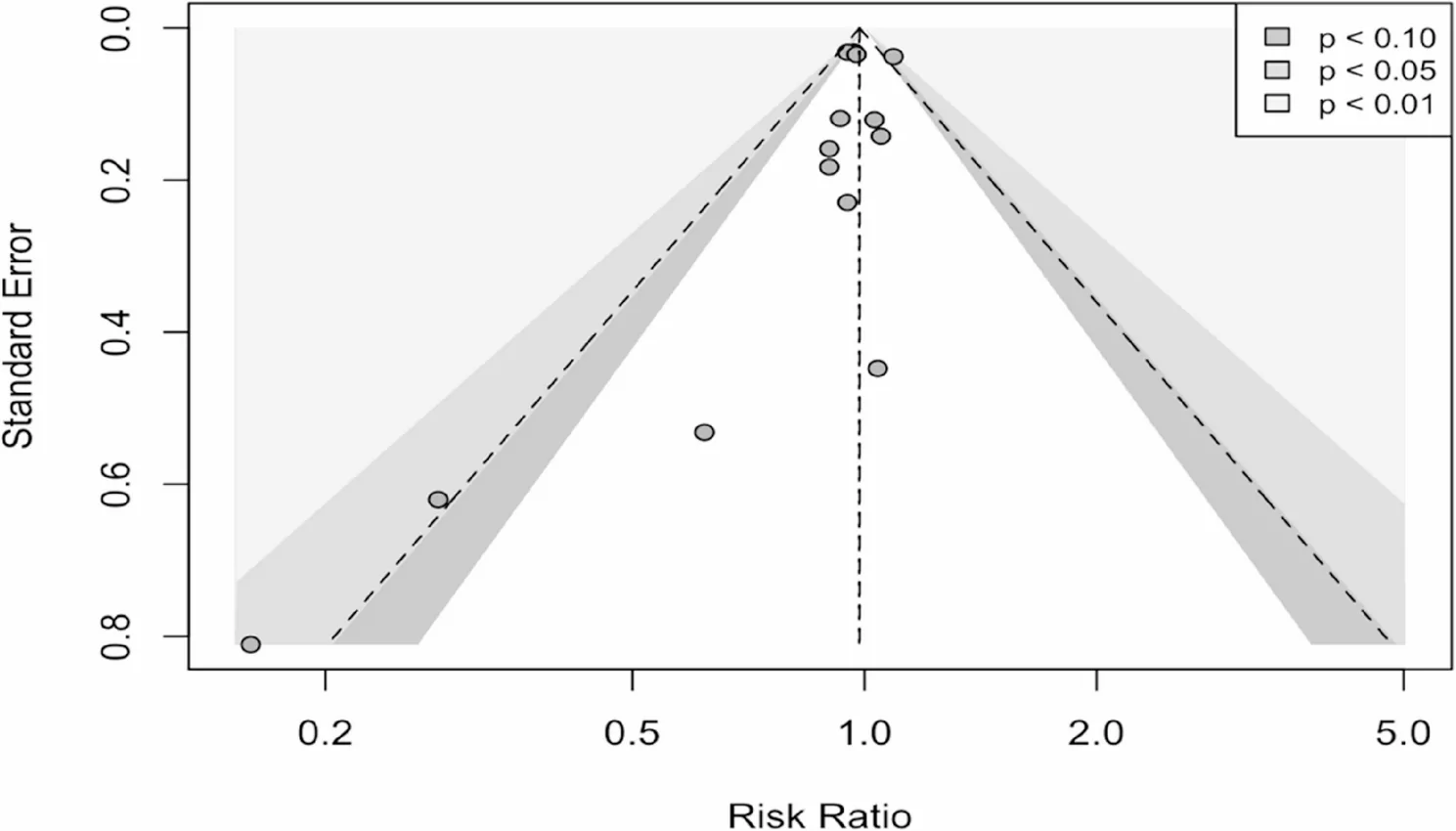
Contour-enhanced funnel plot for the assessment of publication bias. Standard errors of log-transformed risk estimates plotted against effect sizes

The funnel plot (Fig. 3) demonstrated slight visual asymmetry, particularly among smaller studies reporting null or adverse effects, suggesting the possibility of publication bias. This observation was further supported by the contour-enhanced funnel plot (Fig. 4), which showed a relative absence of studies in areas of low statistical significance, a pattern compatible with selective publication. However, Egger’s regression test [39] did not detect statistically significant small-study effects (t = − 1.70; df = 12; *p* = 0.115). Given the limited number of included studies, the statistical power of this test was low, and the result should therefore be interpreted with caution [56, 57]. To further investigate the observed funnel plot asymmetry, a limit meta-analysis and a Q-Q′ test were performed. In the limit meta-analysis, the pooled estimate changed from 0.984 (95% CI 0.930–1.041) to 1.013 (95% CI 0.960–1.070). This shift across the null indicates that the pooled estimate is sensitive to assumptions concerning small-study effects. Furthermore, moderate between-study heterogeneity persisted (I² = 34.8%). The Q-Q′ test suggested that part of the observed heterogeneity could be explained by small-study effects (Q-Q′ = 3.87; df = 1; *p* = 0.0492). After accounting for these effects, the remaining heterogeneity was no longer statistically significant (Q = 16.08; df = 12; *p* = 0.1874). Taken together, although Egger’s regression test was not statistically significant, the consistent findings from the visual inspection of the funnel plots, the Q-Q′ test, and the limit meta-analysis suggest the presence of modest small-study effects. Nevertheless, these effects did not materially alter the overall conclusions of the meta-analysis.

### Risk of bias assessment

The risk of bias was assessed using the ROBINS-E tool [42] across seven domains. Generally, most studies were classified as having at least some concerns or a high risk, with the least concern related to post-exposure interventions, outcome measurement, and missing data. Overall, most studies demonstrated a moderate risk of bias across the ROBINS-E domains, largely reflecting the inherent limitations of observational nutritional epidemiology. No study was considered to have a very high overall risk of bias. The main sources of bias stemmed from residual confounding and exposure assessment, as all studies relied on self-reported dietary intake via FFQs, which can introduce measurement error.

Cohort studies typically showed a lower risk of confounding compared to case-control studies, although residual confounding factors such as lifestyle and screening behaviors may still exist. Some case-control studies, especially those from non-Western regions, were rated as having a high risk due to retrospective exposure assessment and smaller sample sizes. An in-depth evaluation of each study across all seven domains is available in the supplementary material (see Supplements 1).

## Discussion

### Main findings and comparison with existing evidence

Overall, this present meta-analysis found no statistically significant association between high adherence to the Mediterranean diet and prostate cancer risk, with a relative risk of 0.984 (95% CI: 0.930–1.041; *p* = 0.55). The pooled estimate appeared to be influenced by smaller studies with limited adjustment for confounding, indicating potential small-study effects. After adjusting, the null effect was further attenuated, underscoring the absence of a robust association.

This was supported by the significant Q-Q′ test, indicating small-study effects. Meta-regression analyses did not identify study design or geographical region as relevant sources of heterogeneity; however, these findings should be interpreted cautiously given the limited number of studies.

### Comparison with other tumor entities

In contrast to the inconclusive findings for prostate cancer, multiple meta-analyses have reported protective effects of the Mediterranean diet for other cancer types. For example, a reduced risk has been observed for colorectal cancer [19] and breast cancer, including evidence from the PREDIMED (Prevenció con Dieta Mediterránea) randomized trial [16, 58]. Similar associations have been reported in observational studies [20], and in the MCC-Spain study [15].

These differences may reflect biological and methodological factors. Breast cancer is more strongly influenced by hormonal pathways that may be modulated by diet, whereas prostate cancer etiology is more complex and potentially less responsive. Despite the lack of a clear association with incidence, evidence suggests broader health benefits of the Mediterranean diet. Observational studies have reported reduced overall mortality among prostate cancer patients [14] and in general populations [26], while umbrella reviews indicate consistent inverse associations with overall and cancer-specific mortality [6, 12].

Collectively, the literature suggests that the Mediterranean diet may confer broad oncologic and metabolic health benefits, even if its effect on prostate cancer incidence appears weaker.

### Biological plausibility and potential mechanisms

In theory, multiple biological mechanisms support the idea that Mediterranean dietary patterns may protect against cancer development, including prostate cancer. The diet is high in polyphenols, carotenoids, monounsaturated fats, particularly olive oil [59], and dietary fiber, all of which have strong antioxidant and anti-inflammatory properties. Polyphenols and carotenoids can reduce oxidative stress and DNA damage, which are key factors in carcinogenesis [8, 9, 60]. In addition to mitigating oxidative stress and DNA damage, these bioactive compounds may modulate key cancer-related pathways, including phosphoinositide 3-kinase (PI3K)-AKT-mechanistic target of rapamycin (mTOR)-signaling involved in inflammation, cell proliferation, apoptosis, and metabolic signaling. However, these proposed mechanisms provide biological plausibility rather than evidence of a prostate-cancer-specific protective effect [61–63]. Further anti-inflammatory effects involve lowering pro-inflammatory cytokines (Interleukin-6, Tumor necrosis factor-alpha) and inhibiting Nuclear factor-kappa B(NF-κB) activation [64]. Evidence from interventional trials supports the suggested effect on these pathways. For instance, Esposito et al. [65] observed reductions in C-reactive protein (CRP) and Interleukin-6 (IL-6) after short-term Mediterranean diet interventions. Additionally, improved insulin sensitivity and lower hyperinsulinemia may reduce proliferative signals through insulin and Insulin-like growth factor (IGF-1) pathways. The insulin-IGF-1 axis is known to promote prostate cancer cell growth [66], suggesting a mechanistic connection. The diet also positively influences gut microbiota, increasing short-chain fatty acids such as butyrate, which exert anti-tumor effects through epigenetic modifications and consecutive apoptosis [10]. Notably, some of these effects could even work synergistically as an overall dietary pattern rather than as individual components in isolation [60, 67]. In addition to the anti-inflammatory and antioxidant mechanisms discussed above, androgen signaling plays a pivotal role in prostate carcinogenesis. The androgen receptor (AR) regulates prostate cell growth, differentiation, and survival, and its activation by testosterone and particularly dihydrotestosterone is a key driver of both normal prostate physiology and tumor development [68]. Although androgen signaling is important for prostate cancer development and progression, its role is complex and cannot be reduced to a simple relationship between circulating androgen concentrations and prostate cancer risk. Androgen deprivation can inhibit tumor growth in advanced disease, highlighting the dependence of many prostate cancer cells on AR signaling [69]. Furthermore, alterations in AR signaling, including changes in receptor expression, ligand availability, and downstream signaling pathways, may contribute to prostate carcinogenesis and disease progression [70]. Dietary patterns such as the Mediterranean diet could theoretically influence these pathways indirectly through effects on adiposity, insulin sensitivity, inflammation, and metabolic signaling. However, evidence directly linking Mediterranean diet adherence to clinically meaningful alterations in androgen signaling and subsequent prostate cancer risk remains limited. Therefore, this pathway represents a plausible biological hypothesis rather than an established explanation for the epidemiological association observed in the present meta-analysis.

Despite the biological plausibility of a protective effect, several factors may explain why no statistically significant association was observed in the present meta-analysis. First, the contrast in Mediterranean diet adherence between comparison groups may have been insufficient to detect small differences in prostate cancer risk. Second, dietary intake was predominantly assessed using food frequency questionnaires, which are susceptible to measurement error and may attenuate true associations. Third, prostate cancer has a long latency period, making it challenging to capture the cumulative effects of long-term dietary exposure within the available follow-up periods. Furthermore, residual confounding by lifestyle factors, differences in prostate cancer screening practices, genetic susceptibility, and heterogeneity in Mediterranean diet scoring systems may have further diluted any underlying association. Finally, although Mediterranean dietary patterns have been associated with lower levels of inflammation and oxidative stress and with improved metabolic health, the extent to which these effects are relevant to prostate carcinogenesis remains uncertain.

### Comparison with Western dietary patterns and pro-inflammatory diets

Conversely, pro-inflammatory dietary patterns, particularly Western-style diets characterized by high intakes of processed meats, saturated fats, and ultra-processed foods, have been associated with adverse metabolic and inflammatory profiles that may contribute to carcinogenesis. Against this background, the anti-inflammatory and antioxidant properties of the Mediterranean diet provide a biologically plausible rationale for a potential protective effect. However, the current evidence does not demonstrate a statistically significant reduction in prostate cancer risk. Western dietary patterns, characterized by high intakes of red and processed meats, refined carbohydrates, and ultra-processed foods, have been associated with increased prostate cancer risk [71]. It is suggested that certain components of Western dietary patterns could promote inflammation, oxidative stress, and insulin resistance, all of which may facilitate tumor development [72, 73].

Additionally, studies suggest that dietary patterns with high inflammatory potential, measured by the Dietary Inflammatory Index (DII), are strongly associated with prostate cancer risk (RR = 1.75; 95% CI: 1.34–2.28) [74], further supporting the idea that anti-inflammatory diets like the Mediterranean may offer protective benefits.

### Limitations and strengths of this analysis

A key strength of this study is the comparatively large number of included studies, enabling a more comprehensive synthesis than previous analyses. In addition, contemporary meta-analytic methods, including sensitivity and regression-based analyses, were applied to improve robustness. Nevertheless, this meta-analysis is subject to several limitations inherent in nutritional epidemiology. Moderate heterogeneity (I² ≈ 34.8%) likely reflects differences in study design, dietary assessment methods, regional dietary practices, and scoring systems. Furthermore, the quality and standards of certain food groups vary across countries. Variability in exposure assessment, particularly reliance on dietary assessment methods such as food frequency questionnaires (FFQs), may introduce non-differential misclassification, attenuating true associations [23, 24]. Moreover, definitions of Mediterranean diet adherence and confounder adjustment varied across studies, limiting comparability. We used the most comprehensively adjusted estimate reported by each study; however, maximal statistical adjustment does not necessarily correspond to optimal confounding control. Covariates such as BMI, physical activity, metabolic health, and other dietary or lifestyle factors may be interrelated and may potentially represent mediators, proxies, or variables affected by other causes of the outcome.

In the exploratory sensitivity analysis, the pooled effect estimates were broadly comparable between studies using predefined/index-based measures and those using PCA/factor-derived patterns, and there was no statistical evidence of a difference between these subgroups. Nevertheless, heterogeneity was higher among the predefined/index-based studies (I² = 56.0%), whereas no statistical evidence of heterogeneity was detected among the PCA/factor-derived studies. These findings suggest that differences in exposure operationalization may contribute to variability between studies, although the small number of studies within the subgroups and the substantial methodological differences within each category preclude definitive conclusions. Although the exposure-operationalization sensitivity analysis did not identify a statistically significant difference between predefined/index-based and PCA/factor-derived measures, this analysis should be interpreted as exploratory because each category remained heterogeneous with respect to the specific instruments and exposure contrasts used.

While FFQs may introduce some degree of exposure misclassification, the ROBINS-I evaluation did not identify this domain as a systematically high-risk source of bias across the included studies. Accordingly, the absence of a significant association between adherence to a Mediterranean dietary pattern and prostate cancer risk is unlikely to be solely attributable to exposure misclassification.

Statistical procedures followed established recommendations as stated in the methods section. Evidence of publication bias was mixed: while Egger’s test did not provide strong statistical evidence, the Q-Q′ test within the limit meta-analysis indicated that a proportion of the between-study heterogeneity could be explained by small-study effects, though these tests have limited power with small numbers of studies. This pattern suggests that smaller studies may have contributed disproportionately to the pooled effect size. The limit meta-analysis additionally shifted the pooled estimate from 0.984 to 1.013, crossing the null value. These findings do not establish publication bias, as funnel-plot asymmetry may result from heterogeneity, methodological differences, selective reporting, or genuine differences in effect size. Nevertheless, they indicate that small-study effects cannot be excluded and add uncertainty to the pooled estimate. Even though smaller studies are particularly prone to systematic errors, residual confounding cannot be ruled out even in larger, well-adjusted cohort studies. Case–control designs remain vulnerable to recall bias because dietary intake is assessed retrospectively. The design-stratified sensitivity analysis yielded broadly similar pooled estimates for cohort and case-control studies. The pooled estimate was 0.992 (95% CI 0.939–1.048) among cohort studies and 0.969 (95% CI 0.860–1.093) among case-control studies, with no statistically significant difference between study designs (*p* = 0.661). Although heterogeneity was somewhat higher among case-control studies (I² = 50.5%) than among cohort studies (I² = 26.4%), the limited number of studies within each subgroup warrants cautious interpretation. These findings provide no evidence that the overall association was materially driven by study design, but they do not exclude design-related differences given the limited statistical power.

### Implications for practice and research

Although this meta-analysis did not find a significant protective association between adherence to the Mediterranean diet and prostate cancer incidence, the diet’s strong biological rationale, positive metabolic effects, and established benefits for cardiovascular [17, 64, 65] and metabolic conditions like Diabetes [75] underscore its public health and lifestyle relevance. The Mediterranean diet appears feasible for long-term adoption compared to other dietary patterns [76] and may still represent a relevant component of general cancer prevention strategies.

### Conclusion

This meta-analysis did not find a statistically significant association between high adherence to the Mediterranean diet and prostate cancer risk. Future research should use standardized adherence criteria, include population-specific analyses, and incorporate biomarkers and genetic susceptibility factors to better understand causality. Large prospective cohort studies and, if possible, randomized controlled trials (RCTs) are still needed to determine whether associations remain to be identified. The feasibility of such methods has been demonstrated in large dietary intervention trials like PREDIMED [16], indicating that, despite practical challenges, obtaining randomized evidence in this field is achievable. Although randomized controlled trials would provide the highest level of evidence, evaluating prostate cancer incidence as an endpoint would require very large study populations and prolonged follow-up periods, making such trials logistically challenging and resource intensive. Further well-designed prospective studies are needed to determine whether a true protective association exists or to resolve the remaining uncertainty.

### Key messages

This meta-analysis found no statistically significant association between higher versus lower adherence to the Mediterranean diet and prostate cancer risk. Taken together, the present findings should be interpreted as an absence of statistically significant evidence for an association rather than as evidence that a protective association does not exist. The pooled estimate was close to the null, but its confidence interval remains compatible with a modest reduction in prostate cancer risk. Moreover, the interpretation of the pooled estimate is constrained by substantial heterogeneity in the operationalization of the Mediterranean dietary pattern, differences in study design and confounder adjustment, potential exposure misclassification, and possible small-study effects. These limitations may attenuate or otherwise influence observed associations and preclude a definitive causal interpretation. Even though biological plausibility is strong, supported by antioxidant, anti-inflammatory, hormonal, and microbiome-mediated mechanisms, the Mediterranean diet does not convey a protective effect on a pattern-based approach. Future research should include larger, standardized, and well-adjusted cohort studies and, if possible, randomized controlled trials.

From a public health point of view, following a Mediterranean dietary pattern remains a feasible and accessible lifestyle intervention. Adherence may still serve as a simple, low-threshold lifestyle approach with well-known health advantages for cardiovascular health, metabolic diseases, and longevity.

## Supplementary Information

Supplementary Material 1.

## Data Availability

All data generated or analyzed during this study are included in this published article and its supplementary information files. The extracted study-level data and R code used for the analyses are available from the corresponding author upon reasonable request.

## Abbreviations

BMI: Body Mass Index
CI: Confidence Interval
DF: Degrees of Freedom
FFQ: Food Frequency Questionnaire
GLOBOCAN: Global Cancer Observatory
HK: Hartung–Knapp
HR: Hazard Ratio
I²: I-squared statistic
IGF-1: Insulin-like growth factor 1
MeSH: Medical Subject Headings
NF-κB: Nuclear factor-kappa B
OR: Odds Ratio
PI3K-AKT-mTOR: phosphoinositide 3-kinase (PI3K)-AKT-mechanistic target of rapamycin (mTOR) -
PREDIMED: Prevenció con Dieta Mediterránea
PRISMA: Preferred Reporting Items for Systematic Reviews and Meta-Analyses
PSA: Prostate-specific antigen
QM: Test of Moderators
REML: Restricted Maximum Likelihood
ROBINS-E: Risk Of Bias In Non-randomized Studies of Exposure
RCT: Randomized Controlled Trial
RR: Relative Risk
SE: Standard Error
TNF-α: Tumor necrosis factor-alpha
TE: Treatment Effect
τ²: Between-study variance
OR: Odds Ratio
RoB: Risk of Bias
SD: Standard Deviation
UPF: Ultra-Processed Food
WCRF: World Cancer Research Fund
