# Supplementary material for: Mediterranean diet and risk of prostate cancer: a systematic review and meta-analysis of observational studies
Source: BMC Nutr. 2026 Nov 4;12:234. doi: 10.1186/s40795-026-01544-5 (PMC13737873; doi:10.1186/s40795-026-01544-5)
Supplement: Supplementary file 1 [file 40795_2026_1544_MOESM1_ESM.docx]

Supplementary Material

**Supplementary Methods PRISMA 2020 guidelines**

| Section / Item | PRISMA 2020 Requirement | Location in manuscript |
| --- | --- | --- |
| Title | Identify the report as a systematic review / meta-analysis | Title page |
| Abstract | Structured summary of background, objectives, methods, results, conclusions | Abstract |
| Introduction – Rationale | Describe the rationale for the review | Introduction |
| Objectives | Provide explicit statement of objectives (PICO) | Introduction, “Aim of the Meta-Analysis” |
| Methods – Eligibility criteria | Specify inclusion/exclusion criteria | Methods , “Study Selection” |
| Information sources | Specify databases, registers, and last search date | Methods, “Search Strategy” |
| Search strategy | Present full electronic search strategy | Methods, “Search Strategy”, Supplementary methods S2 |
| Selection process | State how studies were selected | Methods, “Search Strategy”, “Study Selection”, PRISMA flowchart (Figure 1) |
| Data collection process | Describe methods of data extraction | Methods, “Data Extraction” |
| Data items | List and define all outcomes, exposures, effect measures | Methods, “Data extraction”, Results, “Study Characteristics” |
| Study risk of bias assessment | Describe methods for bias assessment | Methods, “Risk of Bias Assessment”, Supplementary methods S4 -Risk of bias assessment – ROBINS-E |
| Effect measures | Specify effect measures (RR, CI) | Methods, “Data Extraction” |
| Synthesis methods | Describe methods to synthesize results (REML, Hartung-Knapp, etc.) | Methods, “Statistical Analysis” |
| Reporting bias assessment | Methods used to assess reporting bias (Egger, Funnel) | Methods, “Heterogeneity and Publication Bias” |
| Certainty assessment | Not applicable (no GRADE, observational data only) | – |
| Study selection | Number of studies, PRISMA flowchart | Results, “Study Selection”, PRISMA flowchart (Figure 1) |
| Study characteristics | Characteristics of included studies | Results, “Study Characteristics”, Table 1 |
| Risk of bias in studies | Summary of ROB assessment | Results, “Risk of Bias Assessment”, Supplementary methods S4 -Risk of bias assessment – ROBINS-E |
| Results of individual studies | Report effect sizes per study | Results “Study Characteristics”, Table 1, Forest plot Figure 2 |
| Results of syntheses | Summary of pooled effects, heterogeneity, subgroup analyses | Results, “Meta-Analysis: Mediterranean Diet and Prostate Cancer Risk”, “Heterogeneity”, “Meta-Regression”, “Leave-One-Out Sensitivity Analysis” |
| Reporting bias | Funnel plot, Egger test | Results, “Publication Bias and Limit Meta-Analysis”, Figure 3 |
| Certainty of evidence | Assessment of certainty in the body of evidence for each synthesis assessed | Certainty of evidence was not formally assessed using GRADE but indirectly through risk of bias assessment; Results, “Risk of Bias Assessment” |
| Discussion – Summary of evidence | Main findings, comparison to literature | Discussion, “Main findings and comparison with existing evidence”, “Limitations and strengths of this analysis”, “Implications for practice and research” |
| Registration and protocol | Protocol and registration (not registered) | The review protocol was not prospectively registered |
| Support | Sources of support, funding | Acknowledgments |
| Competing interests | Declaration of conflicts of interest | Formal statement |
| Availability of data | Statement on data/code availability | Suplementary Methods |

# **Supplementary Methods Extended Search Strategy**

Electronic searches were conducted in PubMed (MEDLINE), Web of Science and Scopus from April 2024 database inception to November 2025 to identify studies investigating adherence to the Mediterranean diet and prostate cancer risk. Only articles published in English were considered.

The complete search strategies applied were as follows:

#### PubMed (MEDLINE)

Date of last search: November 2025

(Mediterranean[tiab] OR "Mediterranean diet"[tiab] OR "Mediterranean dietary pattern"[tiab] OR "Mediterranean-style diet"[tiab] OR "Mediterranean diet score"[tiab]) AND (prostate[tiab] OR "prostate cancer"[tiab] OR prostatic[tiab]) AND Humans[Mesh]

#### Scopus

Date of last search: November 2025

TITLE-ABS-KEY("Mediterranean diet" OR "Mediterranean dietary pattern" OR
"Mediterranean-style diet" OR "Mediterranean diet score" OR mediterranean)
AND
TITLE-ABS-KEY("prostate cancer" OR prostate OR prostatic)

Web of Science

("Mediterranean diet" OR "Mediterranean dietary pattern" 
OR "Mediterranean-style diet" OR "Mediterranean diet score" 
OR Mediterranean)
AND
("prostate cancer" OR prostate OR prostatic)

Time span: 1995-2026

Document type: articles

Language: English

The initial literature search identified 504 records, comprising 146 from PubMed, 216 from Web of Science, and 142 from Scopus. After removing duplicates (n=163) and conducting title and abstract screening (exclusion of Meta-analyses, Reviews or Guidelines, n= 80, studies that did not address prostate cancer, n=16, studies that did not address Mediterranean diet or only partial aspects of it, n=76, or neither the one nor the other, n= 130), 33 full-text articles were assessed for eligibility. Of these, 14 studies met all predefined inclusion criteria and were subsequently included in the meta-analysis. Multiple reasons for exclusion could apply to a single study.

**Suplementary Methods Leave-One-Out Sensitivity Analysis**

A leave-one-out analysis was conducted under both fixed- and random-effects models. Removing each study one at a time did not significantly alter the overall effect, with summary RRs consistently between 0.98 and 99. No single study had an outsized influence.

Interestingly, removing Schulpen et al. (Schulpen et al., 2019) resulted in a slight inverse correlation and significantly reduced heterogeneity, with I² = 0.0%. Conversely, excluding other studies yielded heterogeneity ranging from 37.4% to 53.6%. These findings support the robustness of the overall null effect. Detailed results are provided in the supplementary material (Table S1 and Table S2).

**Supplementary Figures and Tables**

This appendix provides supplementary figures and tables that complement the main text of the dissertation. This supplement includes a leave-one-out sensitivity analysis under a random and common-effects model, as well as the corresponding forest plots.

Statistical analyses were conducted in R (version 4.4.2; R Foundation for Statistical Computing, Vienna, Austria) using the packages meta, metafor, metasens, dplyr, stringr, readr, robvis, and CAMAN.

Table S1. Leave-one-out sensitivity analysis of the association between adherence to the Mediterranean diet and prostate cancer incidence (fixed-effects model)

| **Study omitted** | **RR (95% CI)** | **p-value** | **tau²** | **tau** | **I² (%)** |
| --- | --- | --- | --- | --- | --- |
| Omitting Schulpen | 0.9628 [0.9295 - 0.9973] | 0.0348 | 0.0000 | 0.0003 | 0.0 |
| Omitting Ax | 0.9853 [0.9545 - 1.0171] | 0.3605 | 0.0017 | 0.0411 | 39.8 |
| Omitting Bosire | 0.9909 [0.9549 - 1.0282] | 0.6278 | 0.0025 | 0.0496 | 38.8 |
| Omitting Jalipiran | 0.9858 [0.9549 - 1.0176] | 0.3769 | 0.0017 | 0.0408 | 37.5 |
| Omitting Lavalette | 0.9855 [0.9546 - 1.0174] | 0.3697 | 0.0017 | 0.0414 | 39.8 |
| Omitting Muller | 0.9864 [0.9553 - 1.0186] | 0.4035 | 0.0018 | 0.0422 | 39.1 |
| Omitting Russo | 0.9861 [0.9552 - 1.0179] | 0.3867 | 0.0016 | 0.0406 | 19.6 |
| Omitting Kenfield | 0.9979 [0.9618 - 1.0354] | 0.9121 | 0.0019 | 0.0432 | 34.0 |
| Omitting Castello_old | 0.9863 [0.9553 - 1.0183] | 0.3968 | 0.0017 | 0.0415 | 38.8 |
| Omitting Möller | 0.9845 [0.9535 - 1.0166] | 0.3411 | 0.0018 | 0.0430 | 39.4 |
| Omitting Askari | 0.9862 [0.9554 - 1.0180] | 0.3913 | 0.0016 | 0.0405 | 24.2 |
| Omitting Castello_2023 | 0.9845 [0.9535 - 1.0165] | 0.3392 | 0.0018 | 0.0424 | 39.2 |
| Omitting Urquiza_Salvat | 0.9880 [0.9531 - 1.0240] | 0.5079 | 0.0025 | 0.0501 | 39.6 |
| Omitting Lozano_Lorca | 0.9861 [0.9551 - 1.0180] | 0.3882 | 0.0017 | 0.0414 | 39.1 |
| Pooled estimate | 0.9854 [0.9545 - 1.0172] | 0.3625 | 0.0017 | 0.0409 | 34.8 |

Abbreviations: CI, confidence interval; RR, relative risk; I², heterogeneity; τ², between-study variance; τ, square root of τ²

Table S2. Leave-one-out sensitivity analysis of the association between adherence to the Mediterranean diet and prostate cancer incidence (random-effects model)

| **Study omitted** | **RR (95% CI)** | **p-value** | **tau²** | **tau** | **I² (%)** |
| --- | --- | --- | --- | --- | --- |
| Schulpen | 0.9628 [0.9273 - 0.9997] | 0.0482 | 0.0000 | 0.0003 | 0.0 |
| Ax | 0.9840 [0.9274 - 1.0441] | 0.5642 | 0.0017 | 0.0411 | 39.8 |
| Bosire | 0.9864 [0.9182 - 1.0596] | 0.6834 | 0.0025 | 0.0496 | 38.8 |
| Jalipiran | 0.9851 [0.9300 - 1.0436] | 0.5819 | 0.0017 | 0.0408 | 37.5 |
| Lavalette | 0.9845 [0.9275 - 1.0450] | 0.5791 | 0.0017 | 0.0414 | 39.8 |
| Muller | 0.9862 [0.9282 - 1.0478] | 0.6270 | 0.0018 | 0.0422 | 39.1 |
| Russo | 0.9858 [0.9396 - 1.0344] | 0.5297 | 0.0016 | 0.0406 | 19.6 |
| Kenfield | 0.9935 [0.9292 - 1.0623] | 0.8360 | 0.0019 | 0.0432 | 34.0 |
| Castello old | 0.9861 [0.9291 - 1.0466] | 0.6180 | 0.0017 | 0.0415 | 38.8 |
| Möller | 0.9821 [0.9239 - 1.0440] | 0.5322 | 0.0018 | 0.0430 | 39.4 |
| Askari | 0.9861 [0.9378 - 1.0369] | 0.5556 | 0.0016 | 0.0405 | 24.2 |
| Castello_2023 | 0.9821 [0.9246 - 1.0433] | 0.5277 | 0.0018 | 0.0424 | 39.2 |
| Urquiza-Salvat | 0.9845 [0.9166 - 1.0574] | 0.6421 | 0.0025 | 0.0501 | 39.6 |
| Lozano-Lorca | 0.9856 [0.9288 - 1.0460] | 0.6058 | 0.0017 | 0.0414 | 39.1 |
| Pooled estimate | 0.9842 [0.9303 - 1.0412] | 0.5514 | 0.0017 | 0.0409 | 34.8 |

### Abbreviations: CI, confidence interval; RR, relative risk; I², heterogeneity; τ², between-study variance; τ, square root of τ²

Table 2 Legend: Each row presents the pooled relative risk (RR) and 95% confidence interval (CI) from a leave-one-out analysis, in which one study is omitted at a time from the meta-analysis. Results are based on a random-effects model with restricted maximum-likelihood estimation and Hartung–Knapp adjustment (Hartung and Knapp, 2001).

### Table S3. Meta-regression analyses examining potential sources of heterogeneity

| Moderator | β (logRR) | SE | 95% CI | p-value | QM (df=1) | τ² residual | I² residual | R² (%) |
| --- | --- | --- | --- | --- | --- | --- | --- | --- |
| Study design (cohort vs case–control) | 0.034 | 0.065 | −0.084 to 0.153 | 0.573 | 0.32 | 0.0024 | 30.44% | 0.00 |
| Mediterranean region (yes vs no) | −0.027 | 0.059 | −0.143 to 0.089 | 0.652 | 0.20 | 0.0023 | 29.68% | 0.00 |

Abbreviations: β, regression coefficient (log relative risk); SE, standard error; QM, test of moderators; τ², residual between-study variance; I², residual heterogeneity; R², proportion of heterogeneity explained.

Figure S1: Forest plot portraying the changing effect size when omitting individual studies

Legend: relative risks (RR) with 95% confidence intervals of the individual studies, heterogeneity (I2) and pooled estimate (fixed-effects model)

Figure S2: Forest plot portraying the changing effect size when omitting individual studies

Legend: relative risks (RR) with 95% confidence intervals of the individual studies, heterogeneity (I2) and pooled estimate (random-effects model).

Supplementary Material ROBINS-E and bias assessment

**Risk of Bias Assessment for Kenfield et al. (USA, cohort study; Health Professionals Follow-up Study) ROBINS-E domains**

**Domain 1. Bias due to confounding**

The study adjusted for several important potential confounders, including age, total energy intake, body mass index (BMI), physical activity, smoking, and history of prostate-specific antigen (PSA) screening. Additional factors, including diabetes, family history of prostate cancer, multivitamin/supplement use, dairy product intake, and tomato sauce intake, were considered in the analyses where appropriate. The explicit adjustment for PSA screening is an important strength given its strong relevance to prostate cancer detection and diagnosis. Nevertheless, residual confounding cannot be excluded, particularly with respect to socioeconomic characteristics, health-conscious behaviors, and other dietary or lifestyle factors that may not have been fully captured or appropriately measured. Therefore, some concerns remain regarding residual confounding.

**Assessment: Some concerns**

**Predicted direction of bias:** Insufficient information to determine the direction with confidence.

**Domain 2. Bias in selection of participants into the study**

Participants were recruited from the Health Professionals Follow-up Study, resulting in a relatively homogeneous population with high educational and occupational status. Although this restricts the generalizability of the findings to the broader male population, restricted generalizability does not necessarily constitute selection bias within the study. Participants with implausible energy intake, missing baseline information, or prevalent cancer were excluded according to predefined eligibility criteria. The reported response and follow-up were high, with limited evidence that selection into or retention within the study was strongly related to both Mediterranean diet adherence and subsequent prostate cancer risk.

**Assessment: Low risk of bias**

**Domain 3. Bias in classification of exposures**

Mediterranean diet adherence was assessed using a validated 130-item food-frequency questionnaire (FFQ), which was updated repeatedly during follow-up. The reported correlation with detailed dietary records supports the validity of the exposure assessment, and repeated measurements with cumulative averaging reduce the influence of random within-person variation. Nevertheless, dietary assessment using self-reported FFQs remains susceptible to systematic measurement error and misclassification, particularly for specific food groups relevant to Mediterranean diet scoring. The exposure was therefore not measured without error.

**Assessment: Some concerns**

**Predicted direction of bias:** The direction of bias is uncertain because dietary misclassification may arise from several sources and cannot be assumed to be strictly non-differential.

**Domain 4. Bias due to post-exposure interventions**

The study evaluated a naturally occurring dietary exposure rather than an assigned intervention. There is no clear indication that post-exposure interventions or changes in treatment during follow-up, influenced by prior Mediterranean diet adherence, substantially affected the estimated association. The available information therefore provides no important concern regarding bias from post-exposure interventions.

**Assessment: Low risk of bias / no important concern**

**Domain 5. Bias due to missing data**

The study applied explicit criteria for excluding implausible dietary reports and participants with missing baseline information. Follow-up was reported to be highly complete, and there is no indication that missing outcome or covariate information was sufficiently extensive or differentially distributed to substantially bias the reported association.

**Assessment: Low risk of bias**

**Domain 6. Bias in measurement of outcomes**

Prostate cancer diagnoses were confirmed using medical records, with detailed information on tumor characteristics and PSA values available. Mortality ascertainment was additionally supported by the National Death Index and physician adjudication. Outcome ascertainment was therefore based predominantly on objective clinical information and was unlikely to have been substantially influenced by participants’ Mediterranean diet adherence.

**Assessment: Low risk of bias**

**Domain 7. Bias in selection of the reported result**

The investigators reported results for both traditional and alternative Mediterranean diet scores and presented findings for different prostate cancer outcomes. The availability of supplementary analyses reduces concerns regarding selective reporting. However, the study evaluated multiple dietary scores and outcomes, and the available information does not establish whether all analytical decisions were prespecified in a publicly available analysis plan. The possibility of selective emphasis among multiple analyses therefore cannot be completely excluded.

**Assessment: Some concerns**

**Predicted direction of bias:** Insufficient information to determine the direction with confidence.

**Overall ROBINS-E assessment**

The study was judged to have some concerns regarding overall risk of bias, primarily because of potential residual confounding, unavoidable uncertainty in dietary exposure measurement, and the possibility of selective emphasis among multiple analytical choices. Nevertheless, the study has several important methodological strengths, including prospective exposure assessment, repeated dietary measurements, extensive adjustment for relevant confounders including PSA screening, high follow-up completeness, and reliable ascertainment of prostate cancer outcomes.

**Risk of Bias Assessment for Askari et al. (Iran, case-control study) ROBINS-E domains**

**Domain 1. Bias due to confounding**

The authors adjusted for several relevant covariates, including age, body mass index (BMI), energy intake, smoking, and education. However, important potential confounding domains appear to have remained insufficiently addressed, including prostate cancer screening behavior, genetic or familial predisposition, and other dietary and lifestyle factors that may be associated with both Mediterranean diet adherence and prostate cancer risk. In addition, the relatively small sample size limits the precision and stability of multivariable adjustment. Given the potential for substantial residual confounding in a case-control study of dietary exposure, the reported association may not adequately represent the causal effect of Mediterranean diet adherence.

**Assessment: High risk of bias**

**Predicted direction of bias:** Insufficient information to determine the direction with confidence.

**Domain 2. Bias in selection of participants into the study**

Cases were histopathologically confirmed, and controls were recruited from the same clinical setting. This represents an important strength because the use of a common source population can reduce differential selection between cases and controls. Nevertheless, the representativeness of the control group with respect to the exposure distribution in the population giving rise to the cases is not fully clear. If the probability of selection into the control group was related to Mediterranean diet adherence or factors associated with prostate cancer risk, selection bias could have affected the estimated association.

**Assessment: Some concerns**

**Predicted direction of bias:** Insufficient information to determine the direction with confidence.

**Domain 3. Bias in classification of exposures**

Dietary exposure was assessed retrospectively using a validated self-reported FFQ administered by interview. Although the use of a standardized and previously validated dietary instrument is a strength, exposure assessment occurred in the context of a case-control study after diagnosis of prostate cancer. Consequently, cases may have recalled or reported their previous dietary intake differently from controls because of their disease status or awareness of the study hypothesis. This introduces a relevant risk of differential exposure misclassification and recall bias. The magnitude and direction of such bias cannot be reliably established from the available information.

**Assessment: High risk of bias**

**Predicted direction of bias:** Insufficient information to determine the direction with confidence.

**Domain 4. Bias due to post-exposure interventions**

The study was a case-control investigation of a naturally occurring dietary exposure and did not involve an assigned intervention or longitudinal follow-up during which post-exposure interventions could materially modify the estimated effect. There is no clear evidence that interventions occurring after exposure assessment introduced important bias into the reported association.

**Assessment: Low risk of bias / no important concern**

**Domain 5. Bias due to missing data**

There was no indication of substantial missing data relevant to the exposure, outcome, or principal covariates. The reported sample appeared to have been largely complete for the variables included in the analysis. However, the available reporting does not permit a detailed assessment of the mechanism of any missingness.

**Assessment: Low risk of bias**

**Domain 6. Bias in measurement of outcomes**

Prostate cancer was confirmed histopathologically, providing an objective and reliable outcome assessment. Because outcome ascertainment was based on pathological diagnosis rather than subjective assessment, substantial differential outcome measurement according to Mediterranean diet adherence is unlikely.

**Assessment: Low risk of bias**

**Domain 7. Bias in selection of the reported result**

The study reported adjusted odds ratios for the association between Mediterranean diet adherence and prostate cancer. However, the available reporting provides limited information regarding prespecification of the analytical model, selection among potential dietary exposure definitions or cut-offs, and the availability of alternative analyses. This leaves some uncertainty regarding selective reporting or analytical selection, although there is insufficient evidence to conclude that selective reporting materially influenced the reported estimate.

**Assessment: Some concerns**

**Predicted direction of bias:** Insufficient information to determine the direction with confidence.

**Overall ROBINS-E assessment**

The study was judged to have a high overall risk of bias, driven primarily by concerns regarding residual confounding and retrospective classification of dietary exposure. The latter is particularly relevant because cases may have recalled or reported previous dietary intake differently from controls following prostate cancer diagnosis. Although the study benefits from histopathological outcome confirmation, a standardized dietary assessment instrument, and adjustment for several important covariates, these strengths do not fully mitigate the potential for confounding and differential exposure misclassification.

**Risk of Bias Assessment for Bosire et al. (USA, cohort study; NIH-AARP) ROBINS-E domains**

**Domain 1. Bias due to confounding**

The analysis adjusted for several important potential confounders, including age, body mass index (BMI), total energy intake, education, smoking, physical activity, and ethnicity. PSA screening, an important potential confounder in prostate cancer epidemiology, was also accounted for. These adjustments represent important strengths. Nevertheless, residual confounding cannot be completely excluded, particularly with respect to socioeconomic characteristics, health-conscious behaviors, dietary and supplement use, and other lifestyle factors that may not have been fully measured or adequately controlled.

**Assessment: Some concerns**

**Predicted direction of bias:** Insufficient information to determine the direction with confidence.

**Domain 2. Bias in selection of participants into the study**

The NIH-AARP cohort comprised more than 293,000 men aged 50–71 years recruited from the US population. The very large cohort and clearly defined eligibility criteria reduce concerns about selective participation within the source population. However, participation was restricted to a specific age range and the cohort may differ from the general US population with respect to socioeconomic and health-related characteristics. These limitations primarily affect generalizability rather than necessarily indicating substantial selection bias within the estimated association.

**Assessment: Low risk of bias**

**Domain 3. Bias in classification of exposures**

Dietary intake was assessed using a validated food-frequency questionnaire (FFQ), from which an alternate Mediterranean Diet score (aMED) was derived. The use of a standardized and validated FFQ is a strength, but self-reported dietary assessment remains susceptible to measurement error and misclassification. In addition, a single baseline assessment may not fully capture usual long-term dietary intake. If exposure measurement error was predominantly non-differential with respect to subsequent prostate cancer occurrence, it would generally be expected to attenuate associations; however, the direction of bias cannot be established with certainty.

**Assessment: Some concerns**

**Predicted direction of bias:** Probably towards the null, if the misclassification was predominantly non-differential; however, the direction cannot be established with confidence.

**Domain 4. Bias due to post-exposure interventions**

Mediterranean diet adherence was assessed at baseline, and participants were subsequently followed prospectively. Dietary behavior may have changed during the approximately nine-year follow-up, but such changes represent time-varying exposure rather than a post-exposure intervention in the experimental sense. There is no clear evidence that study participation or knowledge of exposure status resulted in systematic interventions that would substantially bias the estimated association.

**Assessment: Low risk of bias / no important concern**

**Domain 5. Bias due to missing data**

Participants with incomplete dietary information were excluded according to the study’s eligibility criteria. Given the very large cohort and reliable follow-up, there is no indication of substantial missing outcome information or differential missingness that would be expected to materially bias the association.

**Assessment: Low risk of bias**

**Domain 6. Bias in measurement of outcomes**

Prostate cancer cases were identified through state cancer registries, providing an objective and highly standardized source of outcome ascertainment. Outcome classification was therefore unlikely to have been substantially influenced by Mediterranean diet adherence.

**Assessment: Low risk of bias**

**Domain 7. Bias in selection of the reported result**

The study reported results from multiple analytical models, including basic and fully adjusted models, and the findings were broadly consistent across specifications. There was no clear indication that the reported result was selectively chosen from a large set of materially different analyses or outcomes.

**Assessment: Low risk of bias**

**Overall ROBINS-E assessment**

The study was judged to have some concerns regarding overall risk of bias, primarily because of potential residual confounding and uncertainty in the classification of dietary exposure. The study otherwise has substantial methodological strengths, including its very large prospective cohort, adjustment for important prostate cancer risk factors including PSA screening, and reliable registry-based outcome ascertainment. The restricted age range and characteristics of the NIH-AARP population primarily limit generalizability rather than constituting substantial internal selection bias.

**Risk of Bias Assessment for Schulpen et al. (Netherlands, cohort study) ROBINS-E domains**

**Domain 1. Bias due to confounding**

The study adjusted for several important potential confounders, including age, body mass index (BMI), total energy intake, alcohol consumption, education, and family history of prostate cancer. Additional variables, including smoking, physical activity, diabetes, height, and tea and coffee consumption, were considered according to their influence on the estimated association. An important limitation is that individual-level PSA screening information was unavailable. PSA screening was addressed indirectly through stratification according to the period before and after the introduction of PSA testing in the Netherlands, but this approach cannot fully account for individual differences in screening behavior. Residual confounding by PSA screening, socioeconomic characteristics, lifestyle, or other unmeasured factors therefore remains possible.

**Assessment: Some concerns**

**Predicted direction of bias:** Insufficient information to determine the direction with confidence.

**Domain 2. Bias in selection of participants into the study**

The cohort included approximately 120,000 participants aged 55–69 years at baseline and was established to represent the Dutch population within the eligible age range. Participants with incomplete dietary or baseline information and those with prevalent cancer were excluded according to defined criteria. The use of a random sub cohort for the estimation of person-years further supports the representativeness of the analytical sample within the source population. The restricted age range limits generalizability to other age groups but does not, by itself, indicate substantial selection bias.

**Assessment: Low risk of bias**

**Domain 3. Bias in classification of exposures**

Dietary intake was assessed at baseline using a validated 150-item FFQ covering intake during the preceding year. However, dietary intake was not reassessed during the approximately 20-year follow-up, creating potential misclassification if dietary habits changed over time. The investigators also calculated multiple Mediterranean diet scores, including aMED and mMED with different treatment of alcohol. Although these measures are relevant representations of Mediterranean diet adherence, their population-specific cut-offs introduce heterogeneity in the classification of exposure across studies rather than necessarily constituting within-study measurement bias.

**Assessment: Some concerns**

**Predicted direction of bias:** The direction is uncertain; if changes in dietary exposure during follow-up were predominantly non-differential, attenuation towards the null would be expected.

**Domain 4. Bias due to post-exposure interventions**

The study assessed a naturally occurring dietary exposure without assigning participants to an intervention. There is no clear evidence that study participation or knowledge of dietary exposure resulted in systematic post-exposure interventions that materially affected prostate cancer incidence. Changes in dietary behavior during follow-up may have occurred, but these primarily represent exposure changes rather than evidence of bias from post-exposure interventions.

**Assessment: Low risk of bias / no important concern**

**Domain 5. Bias due to missing data**

Most covariates had less than 5% missing data, with somewhat greater missingness for smoking frequency. The analyses were based on complete cases. Although complete-case analysis can introduce bias when missingness is associated with exposure and outcome, the relatively low proportion of missing information provides no strong indication of substantial bias.

**Assessment: Low risk of bias**

**Domain 6. Bias in measurement of outcomes**

Prostate cancer outcomes were ascertained through the national cancer registry and the pathology registry (PALGA), with histological confirmation of cases. Outcome ascertainment was therefore objective, highly valid, and unlikely to have been influenced by Mediterranean diet adherence.

**Assessment: Low risk of bias**

**Domain 7. Bias in selection of the reported result**

The investigators reported associations using several dietary scores and presented additional subgroup and sensitivity analyses, including analyses according to PSA-testing era and exclusion of early follow-up. The availability of alternative analyses reduces concerns about selective reporting. Although the observed positive association received particular attention, the study also reported null and alternative findings. There is therefore no strong evidence that the reported result was selectively chosen.

**Assessment: Low risk of bias**

**Overall ROBINS-E assessment**

The study was judged to have some concerns regarding overall risk of bias, primarily because of potential residual confounding, particularly the absence of individual-level PSA screening information, and possible misclassification of dietary exposure resulting from the absence of repeated dietary assessments over the long follow-up period. The study otherwise benefits from a large population-based cohort, clearly defined participant selection, and highly reliable registry-based outcome ascertainment.

**Risk of Bias Assessment for Lavalette et al. (France, cohort study; NutriNet-Santé) ROBINS-E domains**

**Domain 1. Bias due to confounding**

The analyses adjusted for several important potential confounders, including age, sex, education, smoking status, body mass index (BMI), total energy intake, physical activity, and family history of cancer. This represents comprehensive adjustment for several major determinants of prostate cancer risk and dietary behaviour. However, individual-level PSA screening history and some additional socioeconomic and lifestyle factors were not fully accounted for. Residual confounding therefore cannot be excluded.

**Assessment: Some concerns**

**Predicted direction of bias:** Insufficient information to determine the direction with confidence.

**Domain 2. Bias in selection of participants into the study**

The NutriNet-Santé cohort was established through web-based voluntary recruitment. Such recruitment may preferentially include individuals with higher educational attainment, health consciousness, or interest in nutrition. More than 44,000 participants younger than 40 years and approximately 12,000 participants with incomplete information were excluded, resulting in a final analytical population of approximately 41,543 participants. Although these features may limit representativeness and external validity, there is insufficient evidence that selection into the analytical cohort was strongly related to both Mediterranean diet adherence and subsequent prostate cancer risk.

**Assessment: Some concerns**

**Predicted direction of bias:** Insufficient information to determine the direction.

**Domain 3. Bias in classification of exposures**

Dietary intake was assessed repeatedly using validated 24-hour dietary records, with an average of approximately 6.4 assessments over two years. This repeated dietary assessment represents an important strength and is expected to reduce random within-person variation compared with a single dietary assessment. Validation against biomarkers further supports the quality of the exposure measurement. Nevertheless, self-reported dietary assessment remains susceptible to under- and over-reporting, and participants with implausible energy intakes were excluded. The study also used several dietary indices, including MEDI-LITE, although the exposure relevant to the present meta-analysis was based on the prespecified Mediterranean diet measure extracted from the study.

**Assessment: Some concerns**

**Predicted direction of bias:** Insufficient information to determine the direction with confidence.

**Domain 4. Bias due to post-exposure interventions**

The study assessed naturally occurring dietary behaviour and did not assign participants to a dietary intervention. There is no evidence that participation in the cohort systematically induced post-exposure interventions that would materially alter the association between baseline dietary exposure and prostate cancer incidence.

**Assessment: Low risk of bias / no important concern**

**Domain 5. Bias due to missing data**

Missing values for covariates were generally below 5% and were handled using imputation. Participants with implausibly low energy intake were excluded according to predefined criteria. The relatively limited extent of missing information provides no strong indication of substantial bias arising from missing data.

**Assessment: Low risk of bias**

**Domain 6. Bias in measurement of outcomes**

Cancer diagnoses were initially self-reported but subsequently confirmed through medical records for more than 90% of cases. Additional validation was performed using the national health insurance database and mortality registry. This multi-source ascertainment substantially reduces concerns regarding outcome misclassification.

**Assessment: Low risk of bias**

**Domain 7. Bias in selection of the reported result**

The investigators reported associations for several dietary indices, including WCRF/AICR, AHEI-2010, MEDI-LITE, and PNNS-GS. The presence of multiple exposure definitions creates some potential for analytical selection; however, the results for the different scores were reported rather than omitted. Greater discussion of some positive findings than of null findings does not by itself establish selective reporting of the numerical results.

**Assessment: Some concerns**

**Predicted direction of bias:** Insufficient information to determine the direction with confidence.

**Overall ROBINS-E assessment**

The study was judged to have some concerns regarding overall risk of bias, primarily related to residual confounding, the potential for selective emphasis among multiple dietary indices, and the limitations inherent to self-reported dietary exposure assessment. The study nevertheless has important strengths, particularly its repeated dietary assessments, use of validated dietary instruments, relatively low extent of missing data, and extensive validation of cancer outcomes.

**Risk of Bias Assessment for Jalilpiran et al. (Iran, case-control study) ROBINS-E domains**

**Domain 1. Bias due to confounding**

The authors adjusted for several relevant covariates, including age, body mass index (BMI), total energy intake, physical activity, smoking, education, height, and medication use. However, potentially important confounders, including PSA screening history, detailed family history or genetic predisposition, and dietary supplement use, were not adequately addressed. Given the multifactorial determinants of both dietary behaviour and prostate cancer risk, substantial residual confounding remains plausible. The relatively small hospital-based study population further limits the stability and precision of multivariable adjustment, although limited precision itself is not considered evidence of bias.

**Assessment: High risk of bias**

**Predicted direction of bias:** Insufficient information to determine the direction with confidence.

**Domain 2. Bias in selection of participants into the study**

Prostate cancer cases were histologically confirmed and controls were recruited from the same hospital setting, which provides some protection against selection from entirely different source populations. However, hospital-based controls may differ from the population giving rise to the cases with respect to dietary habits and other prostate cancer risk factors. If the probability of selection as a control was associated with Mediterranean diet adherence or factors related to prostate cancer risk, the estimated association could be biased.

**Assessment: Some concerns**

**Predicted direction of bias:** Insufficient information to determine the direction.

**Domain 3. Bias in classification of exposures**

Mediterranean diet adherence was assessed retrospectively through face-to-face interviews using a validated food-frequency questionnaire. Although interviewer administration and use of a validated instrument are strengths, cases were asked to recall dietary behaviour after prostate cancer diagnosis, whereas controls did not have the same disease experience. This creates a substantial possibility of differential recall or reporting of dietary intake between cases and controls. Such differential exposure misclassification may materially affect the estimated association, and its direction cannot be determined with confidence.

**Assessment: High risk of bias**

**Predicted direction of bias:** Insufficient information to determine the direction with confidence.

**Domain 4. Bias due to post-exposure interventions**

The study assessed a naturally occurring dietary exposure and did not assign participants to an intervention. There is no clear evidence that post-exposure interventions or changes induced by study participation materially influenced the reported association.

**Assessment: Low risk of bias / no important concern**

**Domain 5. Bias due to missing data**

Participants with incomplete dietary information were excluded. The study does not provide evidence of substantial missingness in the principal exposure or outcome variables. Although the small sample size means that even limited missing information could reduce precision, there is insufficient evidence that missing data introduced substantial systematic bias.

**Assessment: Low risk of bias**

**Domain 6. Bias in measurement of outcomes**

Prostate cancer cases were confirmed histologically, providing a robust and objective outcome definition. Differential misclassification of the outcome according to dietary exposure is therefore unlikely.

**Assessment: Low risk of bias**

**Domain 7. Bias in selection of the reported result**

Both crude and adjusted associations were reported, and odds ratios were presented transparently. However, the study involved a relatively small number of participants and several potential covariates and analytical choices. The available information does not establish whether the final model and exposure contrast were prespecified or selected from multiple alternative analyses. Therefore, some concerns remain regarding selection of the reported result, although there is insufficient evidence to conclude that selective reporting materially affected the estimate.

**Assessment: Some concerns**

**Predicted direction of bias:** Insufficient information to determine the direction.

**Overall ROBINS-E assessment**

The study was judged to have a **high overall risk of bias**, primarily because of substantial potential for residual confounding and differential misclassification of dietary exposure arising from retrospective assessment after prostate cancer diagnosis. The hospital-based case-control design introduces additional concerns regarding participant selection. Histopathological confirmation of prostate cancer and use of a validated dietary assessment instrument are important strengths, but they do not fully mitigate the potential for confounding and recall bias.

**Risk of Bias Assessment for Russo et al. (Italian case-control study) ROBINS-E domains**

**Domain 1. Bias due to confounding**

**Assessment: Some concerns**

The study adjusted for several important potential confounders, including age, total energy intake, body mass index, smoking, alcohol consumption, physical activity, and family history of prostate cancer. This represents a relatively comprehensive adjustment strategy. However, potentially relevant factors such as socioeconomic circumstances, supplement use, and prostate cancer screening behavior, including PSA testing frequency, were not fully accounted for. Residual confounding therefore remains possible, particularly if these factors are associated with both dietary habits and prostate cancer detection.

**Domain 2. Bias due to selection of participants**

**Assessment: Low**

Prostate cancer cases were histologically confirmed and recruited from a clinical center, while controls were randomly selected from the same geographic area and matched with respect to age, BMI, and smoking. The selection procedures appear appropriate for the case-control design, and there is no clear indication that selection into the study was jointly related to Mediterranean diet adherence and prostate cancer risk. Although recruitment from a single center may limit generalizability, this does not in itself constitute selection bias within the source population.

**Domain 3. Bias due to classification of exposures**

**Assessment: High**

Dietary exposure was assessed retrospectively using a validated FFQ tailored to the Sicilian population. The validation and population-specific adaptation strengthen the exposure assessment; nevertheless, retrospective dietary assessment in a case-control study is particularly susceptible to recall error. Participants with prostate cancer may have recalled or reported their previous dietary habits differently from controls because of their disease status or awareness of dietary risk factors. This creates a plausible risk of differential exposure misclassification. In addition, the derivation of the Mediterranean diet score depends on predefined categorization and scoring decisions, although this aspect primarily contributes to between-study exposure heterogeneity rather than necessarily constituting bias within the study.

**Domain 4. Bias due to post-exposure interventions**

**Assessment: Low / no important concerns**

The study evaluated a naturally occurring dietary exposure and did not assign participants to an intervention. There is no indication that study participation resulted in systematic post-exposure interventions that would have substantially affected the relationship between prior dietary exposure and prostate cancer.

**Domain 5. Bias due to missing data**

**Assessment: Low**

No substantial or systematic missingness was reported for the dietary exposure or other key variables. There were no relevant losses to follow-up because of the case-control design, and the available information suggests that missing data were unlikely to have materially influenced the results.

**Domain 6. Bias in measurement of outcomes**

**Assessment: Low**

The outcome, prostate cancer, was established by histological diagnosis. This provides a highly reliable outcome definition and substantially limits the possibility of outcome misclassification. There is also no apparent reason to expect systematic differential outcome measurement according to Mediterranean diet adherence.

**Domain 7. Bias in selection of the reported result**

**Assessment: Some concerns**

The study reported several analytical specifications, including adjusted and unadjusted models and analyses incorporating additional dietary factors. Multiple analytical choices create some potential for selective emphasis. However, the available information does not provide sufficiently strong evidence that results were selectively chosen for reporting based specifically on their statistical significance. The appropriate judgement is therefore some concerns rather than high risk.

**Overall ROBINS-E assessment**

The overall risk of bias is judged to be high, primarily because of the substantial potential for differential recall and exposure misclassification inherent in the retrospective dietary assessment, together with some concerns regarding residual confounding. Selection and outcome measurement appear comparatively robust, while there is no important concern regarding post-exposure interventions or missing data.

**Risk of Bias Assessment for Muller et al. (Australian cohort study) ROBINS-E domains**

**Domain 1. Bias due to confounding**

**Assessment: Some concerns**

The study adjusted for total energy intake and ethnicity as mandatory covariates, while additional factors including BMI, physical activity, smoking, alcohol consumption, and education were evaluated and did not materially alter the hazard ratios. This indicates consideration of several important confounding factors. Nevertheless, potentially relevant determinants such as family history of prostate cancer, PSA screening behavior, and genetic susceptibility were not comprehensively accounted for. Residual confounding therefore cannot be excluded.

**Domain 2. Bias due to selection of participants**

**Assessment: Low**

The prospective cohort included more than 17,000 men, with 14,627 participants retained in the final analysis. Participants with previous coronary heart disease, diabetes, or prostate cancer and those with extreme energy-intake values were excluded according to predefined eligibility criteria. There is no clear indication that selection into the analytic cohort was jointly related to Mediterranean dietary pattern and subsequent prostate cancer risk. The exclusions may affect generalizability, but they do not by themselves indicate substantial selection bias.

**Domain 3. Bias due to classification of exposures**

**Assessment: Some concerns**

Dietary intake was assessed prospectively using a 121-item FFQ developed for the Melbourne cohort. Factor analysis identified several dietary patterns, including a Mediterranean pattern. Although FFQ-based dietary assessment is subject to measurement error and imperfect classification of dietary patterns, exposure assessment preceded the occurrence of prostate cancer, substantially reducing the likelihood of differential recall related to subsequent disease status. The prospective design therefore makes nondifferential measurement error the principal concern, which would generally be expected to attenuate associations rather than systematically create differential bias.

**Domain 4. Bias due to post-exposure interventions**

**Assessment: Low / no important concerns**

The study assessed naturally occurring dietary patterns and did not assign participants to an intervention. Changes in dietary behavior during follow-up were not systematically captured; however, this represents a limitation of exposure updating rather than evidence of bias due to post-exposure interventions. There is no indication that study participation resulted in differential interventions affecting prostate cancer risk.

**Domain 5. Bias due to missing data**

**Assessment: Low**

A relatively small number of participants were excluded because of missing data, while the majority of exclusions were related to predefined eligibility criteria or implausible energy intake. The reported proportion of participants with missing data was below 1%. There is no clear evidence that missingness was strongly related to both dietary exposure and prostate cancer risk.

**Domain 6. Bias in measurement of outcomes**

**Assessment: Low**

Prostate cancer incidence was ascertained through the Victorian Cancer Registry, a validated population-based cancer registry. Registry-based ascertainment provides standardized outcome measurement and minimizes the risk of differential misclassification according to dietary pattern.

**Domain 7. Bias in selection of the reported result**

**Assessment: Low**

The study reported the principal dietary patterns identified through the factor analysis and additionally presented analyses according to tumor characteristics, Gleason score, and age. The available information does not suggest that results were selectively reported on the basis of their statistical significance. Overall, there are no important concerns regarding selection of the reported result.

**Overall ROBINS-E assessment**

The study is judged to have some concerns regarding risk of bias, primarily due to residual confounding and limitations inherent to FFQ-based dietary exposure measurement. The prospective assessment of diet before cancer diagnosis, large cohort, low proportion of missing data, and reliable registry-based outcome ascertainment substantially strengthen the study.

**Risk of Bias Assessment for** **Castelló et al. (Spanish cohort study) ROBINS-E domains**

**Domain 1. Bias due to confounding**

**Assessment: Some concerns**

The analysis adjusted for several important potential confounders, including age, BMI, total energy intake, education, family history of prostate cancer, and province. Smoking and alcohol consumption were additionally considered in sensitivity analyses. Nevertheless, potentially important determinants such as PSA screening behavior, socioeconomic circumstances beyond education, and physical activity were not comprehensively incorporated into the primary model. Residual confounding therefore remains possible.

**Domain 2. Bias due to selection of participants**

**Assessment: Some concerns**

Prostate cancer cases were histologically confirmed and recruited from 14 Spanish hospitals at first diagnosis, while controls were selected from general practitioner registries. The population-based control recruitment is a strength; however, the participation rate among controls was approximately 52%, raising some concern that participation may have been related to characteristics associated with dietary behavior and prostate cancer risk. Differences between cases and controls in characteristics such as education and alcohol consumption further support some concern regarding selection into the study. However, these differences alone do not establish substantial selection bias.

**Domain 3. Bias due to classification of exposures**

**Assessment: High**

Dietary intake was assessed retrospectively using a validated 154-item FFQ. Because dietary information was collected after prostate cancer diagnosis, differential recall of previous dietary habits between cases and controls is plausible. This concern is compounded by the use of dietary patterns derived through principal component analysis from an external dataset rather than recalculated specifically for the MCC-Spain population. Although the external derivation may improve reproducibility and comparability, it may also result in imperfect classification of adherence to the relevant dietary pattern within the study population. Overall, there is a substantial potential for exposure misclassification.

**Domain 4. Bias due to post-exposure interventions**

**Assessment: Low / no important concerns**

The study investigated naturally occurring dietary exposure and did not assign participants to a dietary intervention. There is no evidence that participation resulted in post-exposure interventions that would have substantially influenced the estimated association between dietary pattern and prostate cancer.

**Domain 5. Bias due to missing data**

**Assessment: Low**

Participants with incomplete FFQs or implausible energy-intake values were excluded. The final analysis included 733 cases and 1,229 controls, while only a relatively small number of participants had missing information (21 cases and 48 controls). There is no clear indication that missingness was strongly related to both exposure and outcome. The potential influence of these exclusions is therefore considered limited.

**Domain 6. Bias in measurement of outcomes**

**Assessment: Low**

Prostate cancer diagnoses were histologically confirmed. Additional clinical characteristics, including Gleason score, tumor stage, and PSA levels, were obtained from medical records. Outcome ascertainment was therefore reliable and standardized, with little concern that outcome measurement depended on dietary exposure.

**Domain 7. Bias in selection of the reported result**

**Assessment: Some concerns**

The study reported overall prostate cancer associations as well as subgroup analyses, including analyses of aggressive disease. The emphasis placed on associations observed for aggressive tumors raises some concern about selective emphasis among multiple analytical results. However, the available information does not establish that statistically significant findings were selectively chosen for reporting. The appropriate judgement is therefore some concerns rather than high risk.

**Overall ROBINS-E assessment**

The overall risk of bias is judged to be high, primarily because the retrospective assessment of dietary exposure creates a plausible risk of differential recall and exposure misclassification, compounded by some concerns regarding participant selection and residual confounding. Outcome ascertainment and missing-data handling appear comparatively robust.

**Risk of Bias Assessment for Möller et al. (Scandinavian/Swedish study) ROBINS-E domains**

**Domain 1. Bias due to confounding**

**Assessment: Some concerns**

The study applied extensive adjustment for potential confounders, including age, region, education, smoking, BMI, total energy intake, physical activity, diabetes, and family history of prostate cancer. This represents a relatively comprehensive confounder strategy. Nevertheless, residual confounding cannot be completely excluded, particularly with respect to PSA screening behavior, socioeconomic factors not fully captured by the available variables, genetic susceptibility, and supplement use. Overall, some concerns remain regarding incomplete control of confounding.

**Domain 2. Bias due to selection of participants**

**Assessment: Some concerns**

Cases were identified through Swedish cancer registries, while controls were selected from the national population register and frequency-matched by age and region. The population-based recruitment strategy is a major strength. However, participation differed between cases (79%) and controls (67%), which raises some concern about differential participation and possible selection bias if participation was associated with both dietary behavior and prostate cancer risk. The difference in participation rates does not by itself demonstrate substantial bias, but warrants a judgement of some concerns.

**Domain 3. Bias due to classification of exposures**

**Assessment: High**

Mediterranean diet adherence was assessed using an MDS derived from a validated 106-item FFQ. Validation against dietary records and 24-hour recalls supports the validity of the instrument, although correlations varied considerably across food groups. Because dietary exposure was assessed retrospectively in the case-control setting, differential recall between cases and controls remains plausible. General limitations of FFQ-based assessment and potential inaccuracies in estimating long-term dietary intake further contribute to uncertainty in exposure classification.

**Domain 4. Bias due to post-exposure interventions**

**Assessment: Low / no important concerns**

The study assessed a naturally occurring dietary exposure and did not assign participants to a dietary intervention. There is no evidence that study participation led to systematic post-exposure interventions that could have substantially altered the association between Mediterranean diet adherence and prostate cancer.

**Domain 5. Bias due to missing data**

**Assessment: Low**

Participants with incomplete dietary information or implausible energy intake were excluded. A total of 94 participants out of 2,590 (approximately 3.6%) were excluded for these reasons. The available information does not suggest substantial or differential missingness among the remaining participants. The potential influence of missing data is therefore considered limited.

**Domain 6. Bias in measurement of outcomes**

**Assessment: Low**

Prostate cancer diagnoses were histologically confirmed and identified through national cancer registries. This provides reliable and standardized outcome ascertainment and minimizes the risk of differential outcome misclassification according to dietary exposure.

**Domain 7. Bias in selection of the reported result**

**Assessment: Low**

The variants of the Mediterranean Diet Score were defined a priori and were reported consistently. The study also provided subgroup analyses and analyses involving different score specifications. Although multiple analytical possibilities inherently create some opportunity for selective emphasis, the available information does not indicate that results were selectively reported according to their statistical significance.

**Overall ROBINS-E assessment**

The overall risk of bias is judged to be high, driven principally by concerns regarding exposure classification and, to a lesser extent, confounding and participant selection. The retrospective dietary assessment creates a plausible risk of differential recall, despite the use of a validated FFQ. Conversely, the population-based control selection, reliable cancer registry ascertainment, and relatively low proportion of excluded participants are important strengths.

**Risk of Bias Assessment for Ax et al. (Swedish cohort study) ROBINS-E domains**

**Domain 1. Bias due to confounding**

**Assessment: Some concerns**

The study adjusted for several important potential confounders, including age, total energy intake, smoking status, physical activity, and educational level. Exclusion of men with pre-existing diabetes and consideration of competing risks of death further strengthened the analytical approach. Nevertheless, residual confounding cannot be completely excluded. In particular, PSA screening behavior, family history of prostate cancer, supplement use, and other health-related or socioeconomic characteristics were not comprehensively accounted for. Dietary patterns may also be correlated with broader health-seeking behaviors that are difficult to capture fully through conventional covariate adjustment. Overall, some concerns remain regarding residual confounding.

**Domain 2. Bias due to selection of participants**

**Assessment: High**

Participants originated from the well-established ULSAM cohort with a clearly defined source population and high initial participation. However, a substantial proportion of participants were excluded because they were classified as inadequate energy reporters, with approximately 46% excluded on this basis. Adequate and inadequate reporters differed systematically in characteristics such as health status and educational attainment. Because reporting adequacy may be associated with dietary behavior and potentially with subsequent prostate cancer risk, these exclusions may have introduced selection bias into the analytic population. This concern extends beyond limited generalizability because the selection of participants for analysis may be related to characteristics associated with both exposure and outcome.

**Domain 3. Bias due to classification of exposures**

**Assessment: Some concerns**

Dietary exposure was assessed using 7-day food records, providing detailed information on food intake and generally offering greater short-term dietary detail than a brief FFQ. The Mediterranean dietary pattern was operationalized using an established modified Mediterranean Diet Score, together with a low-carbohydrate–high-protein score, and energy adjustment was applied. Nevertheless, dietary reporting remains subject to measurement error, and exposure was assessed only at baseline. Furthermore, modifications to the original Mediterranean Diet Score were required because of low consumption of certain food groups, which may have affected construct validity and comparability with other Mediterranean diet scores. Overall, some concerns remain regarding exposure classification, although the prospective nature of the assessment reduces the risk of differential recall by subsequent cancer status.

**Domain 4. Bias due to post-exposure interventions**

**Assessment: Low / no important concerns**

The study assessed naturally occurring dietary patterns rather than an assigned intervention. Consequently, there was no intended dietary intervention from which participants could deviate. Changes in dietary behavior after baseline were not systematically captured, but this represents a limitation of exposure updating rather than evidence of bias due to post-exposure interventions. There is no indication that study participation resulted in systematic interventions affecting subsequent prostate cancer risk.

**Domain 5. Bias due to missing data**

**Assessment: Low**

Missing information for individual covariates such as smoking and physical activity reduced the number of participants available for some fully adjusted models, but the proportion of missing data was relatively small. Participant numbers were clearly reported for the different models, and outcome data were available through national cancer and mortality registries. There is no clear evidence that missingness was strongly related to both dietary exposure and subsequent prostate cancer risk. Overall, missing data are unlikely to have substantially biased the results.

**Domain 6. Bias in measurement of outcomes**

**Assessment: Low**

Prostate cancer cases were identified through the Swedish Cancer Register, which provides comprehensive population-based ascertainment. Outcome classification was objective and independent of dietary exposure assessment. Additional information on tumor characteristics was obtained from medical records for relevant subgroup analyses. There is therefore little concern regarding differential outcome misclassification.

**Domain 7. Bias in selection of the reported result**

**Assessment: Low**

The study reported analyses based on predefined dietary pattern scores and clearly described the statistical models used. Additional analyses, including analyses restricted to adequate energy reporters and models incorporating selenium intake, were transparently presented. There is no clear indication that results were selectively chosen for reporting according to their statistical significance.

**Overall ROBINS-E assessment**

The study is judged to have an overall high risk of bias, driven primarily by potential selection bias arising from the exclusion of approximately 46% of participants classified as inadequate energy reporters. Additional concerns relate to residual confounding and limitations in dietary exposure measurement. Important strengths include the prospective design, detailed dietary assessment, reliable registry-based outcome ascertainment, and transparent reporting.

**Risk of Bias Assessment for Lozano-Lorca et al. (Spanish case-control study) ROBINS-E domains**

**Domain 1. Bias due to confounding**

**Assessment: Some concerns**

The authors adjusted their multivariable models for several important potential confounders, including age, total energy intake, educational level, family history of prostate cancer, and body mass index. These variables capture several major determinants of both dietary behavior and prostate cancer risk. However, potentially relevant factors such as PSA screening behavior, supplement use, comorbidities, physical activity, smoking, and socioeconomic circumstances beyond education were not comprehensively accounted for. Given the complex clustering of lifestyle behaviors, residual confounding therefore remains possible.

**Domain 2. Bias due to selection of participants**

**Assessment: Low**

Prostate cancer cases were incident and histologically confirmed, while controls were population-based and recruited from the same geographic catchment areas as the cases. Controls were frequency-matched according to age, and the inclusion and exclusion criteria were clearly described. There is no clear indication that selection into the study was jointly related to dietary exposure and prostate cancer risk. The population-based control strategy is therefore considered appropriate, and there are no important concerns regarding selection bias.

**Domain 3. Bias due to classification of exposures**

**Assessment: High**

Dietary intake was assessed retrospectively using a validated FFQ, and dietary patterns were derived using principal component analysis. Although PCA-based patterns can capture overall dietary behavior, their derivation involves analytical choices concerning food grouping, factor extraction, and interpretation. More importantly, dietary exposure was assessed retrospectively after prostate cancer diagnosis. Consequently, cases may have recalled or reported their previous dietary habits differently from controls, creating a plausible risk of differential exposure misclassification. Overall, the retrospective assessment of dietary exposure represents a substantial source of potential bias.

**Domain 4. Bias due to post-exposure interventions**

**Assessment: Low / no important concerns**

The study investigated naturally occurring dietary exposure and did not assign participants to a dietary intervention. There is no evidence that participation in the study resulted in systematic changes or interventions that would have substantially altered the association between previous dietary behavior and prostate cancer.

**Domain 5. Bias due to missing data**

**Assessment: Low**

The proportion of missing dietary and covariate information was relatively small. Participants with incomplete questionnaires or implausible energy intake were excluded according to predefined criteria, and the handling of missing information was described transparently. There is no clear indication that missingness differed substantially between cases and controls in a manner related to both dietary exposure and prostate cancer risk.

**Domain 6. Bias in measurement of outcomes**

**Assessment: Low**

Prostate cancer diagnoses were confirmed histologically, and additional clinical information, including tumor grade and stage, was obtained from medical records. Outcome ascertainment was therefore objective and standardized and was independent of dietary exposure classification. The risk of differential outcome misclassification is consequently considered low.

**Domain 7. Bias in selection of the reported result**

**Assessment: Some concerns**

The study included multiple analyses, including stratification by tumor aggressiveness and stage. Furthermore, the derivation of dietary patterns using PCA involves several analytical choices that could potentially generate different results. However, the main findings were clearly presented and there is no specific evidence that outcomes or analyses were selectively omitted because of their statistical significance. The available information therefore supports some concerns rather than high risk of selective reporting.

**Overall ROBINS-E assessment**

The study is judged to have an overall high risk of bias, primarily because of the potential for differential recall and exposure misclassification associated with retrospective dietary assessment in a case-control setting. Some concerns also remain regarding residual confounding and analytical flexibility in the derivation of dietary patterns. Population-based control selection, reliable outcome ascertainment, and relatively complete data are important strengths.

**Risk of Bias Assessment for Urquiza-Salvat et al. (Spanish case-control study) ROBINS-E domains**

**Domain 1. Bias due to confounding**

**Assessment: High**

The primary regression models adjusted for age and family history of prostate cancer, both established determinants of prostate cancer risk. However, several potentially important confounders, including total energy intake, socioeconomic circumstances, PSA screening behavior, physical activity, smoking, and BMI, were not consistently included in the final multivariable models. Because these factors are closely associated with dietary behavior and may also influence prostate cancer risk or detection, substantial residual confounding is plausible. The relatively limited confounder adjustment is therefore considered a potentially important threat to the validity of the estimated association.

**Domain 2. Bias due to selection of participants**

**Assessment: High**

Participants were recruited from a hospital-based urological setting among men undergoing prostate biopsy because of elevated PSA levels or clinical suspicion. Controls were biopsy-negative men recruited from the same clinical context. Although this provides a comparable diagnostic pathway and reduces some differences in healthcare access, the study population represents men undergoing prostate evaluation rather than the general population. In particular, the dietary characteristics of biopsy-negative controls may differ systematically from those of men in the source population who were not referred for biopsy. Because the factors leading to biopsy may themselves be related to lifestyle and dietary behavior, selection into the study may be related to both exposure and outcome status. This creates a substantial concern for selection bias.

**Domain 3. Bias due to classification of exposures**

**Assessment: High**

Dietary exposure was assessed retrospectively using a validated Mediterranean Diet Score administered through face-to-face interviews by trained dietitians. The standardized interview procedure strengthens the consistency of exposure assessment; however, the information remained self-reported and retrospective. In a case-control setting, participants with prostate cancer may recall or report previous dietary habits differently from biopsy-negative controls, creating a plausible risk of differential recall and exposure misclassification. The use of a validated score does not eliminate this concern because validation primarily addresses measurement validity and does not prevent differential recall associated with disease status.

**Domain 4. Bias due to post-exposure interventions**

**Assessment: Low / no important concerns**

The study assessed habitual dietary exposure without assigning participants to an intervention. There is no evidence that study participation resulted in systematic dietary interventions or other post-exposure changes that would have materially affected the estimated association.

**Domain 5. Bias due to missing data**

**Assessment: Low**

The study reported complete dietary and clinical information for most participants, with no substantial evidence of differential missingness between cases and controls. There is no clear indication that missing data were jointly related to dietary exposure and prostate cancer status in a manner likely to substantially bias the results.

**Domain 6. Bias in measurement of outcomes**

**Assessment: Low**

Prostate cancer diagnosis was based on histopathological examination following biopsy, providing an objective and standardized outcome definition. Outcome ascertainment was independent of dietary exposure assessment, and there is little concern regarding differential outcome measurement.

**Domain 7. Bias in selection of the reported result**

**Assessment: Low**

The main outcomes and analytical approaches were clearly described, and there is no specific evidence of selective reporting of favorable or statistically significant results. Although secondary analyses were performed, the available information suggests that reporting was sufficiently transparent.

**Overall ROBINS-E assessment**

The study is judged to have an overall high risk of bias, primarily because of substantial concerns regarding confounding, participant selection, and retrospective exposure classification. The hospital-based recruitment of men undergoing prostate biopsy may result in a selected study population, while the limited adjustment for important lifestyle and screening-related factors leaves considerable potential for residual confounding. In contrast, outcome ascertainment and data completeness appear robust.

**Risk of Bias Assessment for Castelló et al. (2023 EPIC-Spain prospective cohort study) ROBINS-E domains**

**Domain 1. Bias due to confounding**

**Assessment: Some concerns**

The study adjusted for a broad range of established and plausible determinants of prostate cancer risk and dietary behavior. Cox proportional hazards models were stratified by study center and age and additionally adjusted for factors including total energy intake, educational level, height, BMI, smoking status, physical activity, and other lifestyle characteristics. These measures substantially reduce the potential for confounding. Nevertheless, residual confounding cannot be completely excluded, particularly with respect to genetic susceptibility, PSA screening behavior, family history of prostate cancer, and healthcare access or utilization that may not have been fully captured. Overall, some concerns remain regarding residual confounding.

**Domain 2. Bias due to selection of participants**

**Assessment: Some concerns**

Participants were recruited into the EPIC-Spain cohort before the occurrence of the outcome, and predefined exclusion criteria were applied consistently. These included exclusions related to implausible energy intake, extreme BMI values, and the restriction of the outcome definition to clinically relevant prostate cancer. Cases diagnosed or censored during the first year of follow-up were also excluded to reduce the possibility of reverse causation. Although some participants were excluded because of missing covariate information, the exclusions were transparently reported and the remaining cohort was large. Overall, there are some concerns that the cumulative exclusions and analytic restrictions could have introduced selection related to participant characteristics, but there is no strong evidence of substantial selection bias.

**Domain 3. Bias due to classification of exposures**

**Assessment: Some concerns**

Western, Prudent, and Mediterranean dietary patterns were derived using a posteriori statistical methods from dietary history questionnaires administered at baseline, before the occurrence of prostate cancer. The use of validated dietary assessment instruments and the prospective timing of exposure measurement reduce concerns regarding differential recall. However, a posteriori dietary pattern derivation involves analytical decisions concerning food grouping, pattern extraction, and interpretation. In addition, the patterns were originally derived in another EPIC population, which may limit the precision with which the resulting patterns represent dietary behavior in the EPIC-Spain population. Dietary changes during follow-up may also have resulted in some exposure misclassification. Overall, these considerations support some concerns regarding exposure classification.

**Domain 4. Bias due to post-exposure interventions**

**Assessment: Some concerns**

As an observational cohort study, no dietary intervention was assigned. However, dietary patterns were measured only at baseline, and participants may have changed their dietary behavior during the follow-up period. Such changes could result in misclassification of long-term exposure. There is no clear evidence that these changes were systematically induced by study participation or related to subsequent prostate cancer development, so the concern is limited. Nevertheless, because exposure status was not repeatedly updated, some concern remains regarding deviations from the baseline exposure trajectory.

**Domain 5. Bias due to missing data**

**Assessment: Low**

Some participants were excluded from adjusted analyses because of missing covariate information, including variables such as education, height, and Gleason grade. The number of exclusions was reported transparently, and the remaining cohort retained a large sample size and substantial numbers of prostate cancer cases. There is no clear evidence that missingness was strongly related to both dietary exposure and prostate cancer incidence. Overall, missing data are unlikely to have substantially biased the results.

**Domain 6. Bias in measurement of outcomes**

**Assessment: Low**

Prostate cancer cases were identified through reliable cancer registries and medical record linkage, with histopathological confirmation and detailed classification according to Gleason and ISUP grade. The outcome definition was therefore objective, standardized, and independent of dietary exposure assessment. Exclusion of non-clinically relevant tumors further increased specificity for the outcome definition used in the analysis.

**Domain 7. Bias in selection of the reported result**

**Assessment: Low**

The statistical analysis was clearly described, and results were reported for the predefined dietary patterns and relevant outcome categories, including overall and stratified analyses according to tumor aggressiveness. There is no evidence of outcome switching or selective omission of null findings. In particular, null findings for the Mediterranean and Prudent patterns were reported alongside the findings for the Western pattern. Overall, there are no important concerns regarding selection of the reported result.

**Overall ROBINS-E assessment**

The study is judged to have some concerns regarding risk of bias, primarily due to residual confounding, potential exposure misclassification from baseline-only dietary assessment and posteriori pattern derivation, and some uncertainty surrounding participant exclusions and missing covariate information. The prospective design, large cohort, comprehensive confounder adjustment, reliable outcome ascertainment, and transparent reporting substantially strengthen the validity of the findings.
